# Mfn2/Hsc70 Complex Mediates the Formation of Mitochondria‐Lipid Droplets Membrane Contact and Regulates Myocardial Lipid Metabolism

**DOI:** 10.1002/advs.202307749

**Published:** 2024-02-04

**Authors:** Lang Hu, Daishi Tang, Bingchao Qi, Dong Guo, Ying Wang, Jing Geng, Xiaoliang Zhang, Liqiang Song, Pan Chang, Wensheng Chen, Feng Fu, Yan Li

**Affiliations:** ^1^ Department of Cardiology Tangdu Hospital Airforce Medical University Xi'an 710032 China; ^2^ Digestive System Department Shaanxi Provincial Crops Hospital of Chinese People's Armed Police Force Xi'an 710032 China; ^3^ Department of Respirology Xijing Hospital Airforce Medical University Xi'an 710032 China; ^4^ Department of Cardiology The Second Affiliated Hospital of Xi'an Medical College Xi'an 710032 China; ^5^ Department of Cardiovascular Surgery Xi'an Gaoxin Hospital Xi'an 710032 China; ^6^ Department of Physiology and Pathophysiology Airforce Medical University Xi'an 710032 China

**Keywords:** Hsc70, lipid overload, Mfn2, mitochondrion‐lipid droplets membrane contacts, myocardial lipotoxicity

## Abstract

The heart primarily derives its energy through lipid oxidation. In cardiomyocytes, lipids are stored in lipid droplets (LDs) and are utilized in mitochondria, although the structural and functional connections between these two organelles remain largely unknown. In this study, visible evidence have presented indicating that a complex is formed at the mitochondria‐LD membrane contact (MLC) site, involving mitochondrion‐localized Mfn2 and LD‐localized Hsc70. This complex serves to tether mitochondria to LDs, facilitating the transfer of fatty acids (FAs) from LDs to mitochondria for β‐oxidation. Reduction of Mfn2 induced by lipid overload inhibits MLC, hinders FA transfer, and results in lipid accumulation. Restoring Mfn2 reinstates MLC, alleviating myocardial lipotoxicity under lipid overload conditions both in‐vivo and in‐vitro. Additionally, prolonged lipid overload induces Mfn2 degradation through the ubiquitin‐proteasome pathway, following Mfn2 acetylation at the K243 site. This leads to the transition from adaptive lipid utilization to maladaptive lipotoxicity. The experimental findings are supported by clinical data from patients with obesity and age‐matched non‐obese individuals. These translational results make a significant contribution to the molecular understanding of MLC in the heart, and offer new insights into its role in myocardial lipotoxicity.

## Graphical abstract

This study is designed to investigate the molecular basis of membrane contact between lipid droplet (LD) and mitochondria. Mitochondrion‐localized Mfn2 directly interacts with LD‐localized Hsc70, facilitating fatty acids (FAs) transferring from LDs to mitochondria for oxidation. Lipid overload‐induced Mfn2 degradation impedes FAs transferring, leading to intra‐myocardial lipid accumulation and maladaptive lipotoxicity.

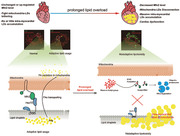

## 1 Introduction

Obesity is an escalating global public health concern.^[^ ^1^ ^]^ Findings from population‐based research underscore the strong correlation between an elevated body mass index (BMI) and heightened morbidity from cardiovascular disease (CVD).^[^ ^2^, ^3^ ^]^ Excessive lipid supply stands out as a prevalent cause of obesity‐related CVD.^[^ ^4^ ^]^ Moreover, fatty acids (FAs) contribute to over 70% of cardiac adenosine triphosphate (ATP) production^[^ ^5^ ^]^ A healthy heart can adapt by enhancing lipid catabolism capacity in response to a short‐term surplus of lipid supply, thereby averting intramyocardial lipid accumulation.^[^ ^6^ ^]^ Conversely, individuals with obesity frequently exhibit abnormal intramyocardial lipid accumulation, leading to impaired left ventricular (LV) diastolic function and increased LV mass.^[^ ^7^, ^8^ ^]^ Despite these observations, the endogenous mechanism orchestrating the shift from adaptive lipid utilization in healthy individuals to maladaptive lipotoxicity in obesity remains poorly understood.

Lipid droplets (LDs) are dynamic organelles comprising phospholipid monolayers surrounding a central core containing predominantly triglycerides (TAG) and cholesteryl ester.^[^ ^9^ ^]^ FAs released from LDs through lipolysis are subsequently utilized in the mitochondria for ATP generation via β‐oxidation. Tight structural and functional connections between LDs and mitochondria appear to play a crucial role in metabolic regulation.^[^ ^10^, ^11^ ^]^ This LD‐mitochondria connection not only facilitates the delivery of FAs but also mitigates cytosolic free FA content, preventing lipotoxicity and abnormal lipid signaling.^[^ ^12^ ^]^ Endurance exercise training has been shown to increase the proportion of LDs in physical contact with mitochondria in human muscle tissue,^[^ ^13^ ^]^ indicating a significant LD‐mitochondrion structural‐functional relationship during high‐energy‐consuming processes. Given the heart's status as one of the most energy‐demanding organs, a favorable coupling between LDs and mitochondria is crucial^[^ ^14^ ^]^ However, the specific molecular basis connecting LDs with mitochondria in cardiomyocytes and its physiological role in cardiac lipid metabolism remains largely unknown.

Mitofusin2 (Mfn2), a transmembrane (TM) protein localized to the outer mitochondrial membrane, is highly conserved from *Drosophila* to humans.^[^ ^15^ ^]^ Mfn2 comprises an N‐terminal GTPase domain and a bipartite TM region that spans the outer mitochondrial membrane twice.^[^ ^16^ ^]^ The unique structure of Mfn2 enables it to induce membrane docking by connecting to the lipid bilayer structure.^[^ ^16^ ^]^ Recent studies have unveiled Mfn2's involvement in membrane contact between organelles, such as the mitochondria‐endoplasmic reticulum (ER) interface and autophagosome‐lysosome fusion.^[^ ^17^, ^18^ ^]^ In the hearts of obese *db*/*db* mice, Mfn2 is significantly decreased, accompanied by intra‐myocardial LD accumulation,^[^ ^19^ ^]^ suggesting Mfn2's potential role in cardiac lipid metabolism. However, the precise function of Mfn2 in lipid metabolism regulation in the heart, particularly under conditions of lipid overload, remains largely unknown.

This study aimed to explore the role of mitochondria‐LD membrane contact (MLC) and the underlying mechanisms in vivo during the transition of lipid metabolism. We demonstrated that mitochondrion‐localized Mfn2 directly interacts with LD‐localized heat shock cognate protein 70 (Hsc70) through its 649–692 amino acid (aa) domain, facilitating MLC formation and promoting FA transfer from LDs to the mitochondria for oxidation in cardiomyocytes. In the early stages of lipid overload, Mfn2 expression is upregulated at both the transcriptional and protein levels in response to increased lipid supply, resulting in unchanged MLC and adaptive lipid utilization in cardiomyocytes. However, as lipid overload persists, accumulated lipid metabolites induce Mfn2 acetylation at a specific lysine residue (K243), leading to its degradation via the ubiquitin‐proteasome pathway. Consequently, Mfn2‐Hsc70‐mediated MLC is disrupted, hindering FA oxidation and triggering maladaptive lipotoxicity.

## 2 Results

### 2.1 Lipid Accumulation and Reduced Mfn2 Levels are Evident in The Hearts of Obese Mice

**Figure 1:**
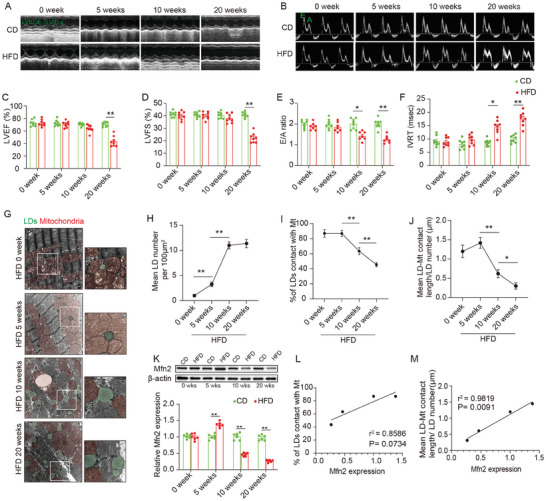
Lipid accumulation, reduced MLC and decreased Mfn2 expression were observed in the heart of obese mice. A–F) Echocardiographic assessment were performed on CD‐ and HFD‐fed WT mice. n = 8 mice per group. A&B, Representative images of M‐mode echocardiography (A) and representative Doppler flow measurement of mitral inflow (B). C–F, Left ventricle ejection fraction (LVEF), left ventricle fractional shortening (LVFS), E/A ratio and isovolumic relaxation time (IVRT). (Mean± SEM, **P<0.01). G) Representative electron microscopy images of LDs and mitochondria in CD‐ and HFD‐fed mice heart. LDs are labeled as green while mitochondria are labeled as red. Scale bars, 1 µm. H) LDs diameter length quantifications from TEM images (Mean± SEM, n = 10 images per group, **P<0.01). I) Intra‐myocardial LD number were calculated to assess lipid accumulation in myocardium (Mean± SEM, n = 10 images per group, **P<0.01). J) Mean LD‐mitochondria (Mt) contact length was measured to reflect LD‐mitochondria tethering in myocardium (Mean± SEM, n = 10 images per group, *P<0.05, **P<0.01). K) Representative blot image and quantification of Mfn2 in hearts of CD‐ or HFD‐fed mice at different time points (Mean± SEM, n = 6 mice per group, **P<0.01). L&M) Pearson's correlation analysis was carried out to evaluate the correlation between Mfn2 expression and indicators for mitochondria‐LDs contact. CD, chow diet; HFD, high‐fat diet; LD, lipid droplet.

Wild‐type (WT) C57BL mice were subjected to either a high‐fat diet (HFD) or a chow diet (CD), and cardiac function and intra‐myocardial lipid accumulation were subsequently examined. After 5 weeks of feeding, HFD resulted in a significantly greater increase in body weight compared to CD, with the disparity continuing to grow as the feeding regimen progressed (Figure S1A, Supporting Information). Serial echocardiography was conducted to monitor cardiac function in both CD‐ and HFD‐fed mice. **Figure** **1**B–F illustrates that, relative to CD‐fed mice, cardiac systolic function (ejection fraction and fractional shortening) and diastolic function (E/A ratio and isovolumic relaxation time) did not exhibit significant changes in HFD‐fed mice after 5weeks of feeding. However, after 10 weeks of feeding, cardiac diastolic function started to significantly decline in HFD‐fed mice, while cardiac systolic function remained unaffected (Figure 1A–F). Subsequently, after 20 weeks of HFD feeding, both systolic and diastolic functions were significantly reduced in HFD‐fed mice compared to their CD‐fed counterparts (Figure 1A–F). Blood biochemistry analysis revealed that fasting plasma glucose, TAGs, total cholesterol, high‐density lipoprotein, and low‐density lipoprotein levels were significantly elevated in HFD‐fed mice after 20 weeks (Figure S1B–F Supporting Information).

Electron microscopy was employed to investigate intramyocardial lipid accumulation. Figure 1G–K illustrates that LDs, labeled in green, were rarely observed in the basal state (CD or HFD at week 0). Following 5 weeks of HFD feeding, a modest number of LDs started appearing in the myocardium, primarily in association with mitochondria. By the 10^th^ week of HFD feeding, intramyocardial LD accumulation became more pronounced, with increased LD numbers in the hearts of mice fed HFD for 10 weeks compared to those fed for 5 weeks (Figure 1G–H). Additionally, MLC was significantly reduced, as evidenced by the decrease in the percentage of LDs in contact with mitochondria (labeled in red) and a shorter MLC length (Figure 1G,I–J). Prolonged HFD feeding (20 weeks) further intensified intramyocardial LD accumulation, leading to an exacerbation of MLC reduction. Notably, segregated LDs with no connection to the mitochondria were commonly observed in the hearts of mice fed an HFD for 20 weeks.

Furthermore, considering the potential role of Mfn2 in lipid metabolism,^[^ ^19^ ^]^ we assessed Mfn2 protein levels in the hearts of HFD‐fed mice. As depicted in Figure 1K, Mfn2 levels exhibited a slight increase in the early stages of HFD feeding (0–5 weeks), followed by a significant decrease after long‐term HFD feeding (10–20 weeks). Additionally, a Pearson correlation analysis was conducted to assess the correlation between Mfn2 levels and indicators for MLC. Figure 1L–M shows a significant correlation between Mfn2 and mean MLC length (*P* = 0.009), with a near‐significant correlation observed between Mfn2 and the percentage of LDs in contact with mitochondria (*P* = 0.07). In summary, these findings suggest an augmentation of intramyocardial LD accumulation and a reduction in LD‐mitochondria connectivity in lipotoxic myocardium, phenomena associated with diminished Mfn2 levels.

### 2.2 Mfn2 Restores MLC and Facilitates The Transfer of FAs from LDs to The Mitochondria

Given that long‐chain FAs are the primary fuel source for the heart, palmitate (Pal) was utilized in in‐vitro experiments to simulate the effects of HFD on the heart. To determine the optimal concentration, cardiomyocytes were incubated with varying concentrations of Pal (0, 50, 150, 300, 500 µM). As depicted in Figure S2A,B (Supporting Information), both 300 µM and 500 µM Pal induced significant intracellular LD accumulation in cardiomyocytes, with significant cytotoxicity observed in the 500 µM group but not the 300 µM group. Therefore, 300 µM Pal was chosen as an effective yet non‐toxic concentration for subsequent in vitro experiments. The impact of lipid overload‐induced myocardial lipotoxicity was further investigated in primary cardiomyocytes in the presence or absence of Pal. MLC was assessed by fluorescently labeling the mitochondria (red) and LDs (green) in Pal‐treated cardiomyocytes. Fluorescence imaging results demonstrated that cells cultured in the control medium (Pal 0 h) exhibited few LDs (Figure S2C,D, Supporting Information), with over 70% of LDs connecting to mitochondria (Figure S2C,E, Supporting Information). In cells treated with Pal for 12 h, the LD area increased while MLC decreased, and these effects were further exacerbated with 24 or 48 h Pal treatments (Figure S2C–E, Supporting Information). Blot analysis results revealed a reduction in Mfn2 protein levels after 12 h Pal treatment, with a more notable decrease observed after 24 h Paltreatment (Figure S2F–G, Supporting Information). These findings suggest that excessive lipid overload hinders MLC formation and diminishes Mfn2 levels in cardiomyocytes.

**Figure 2:**
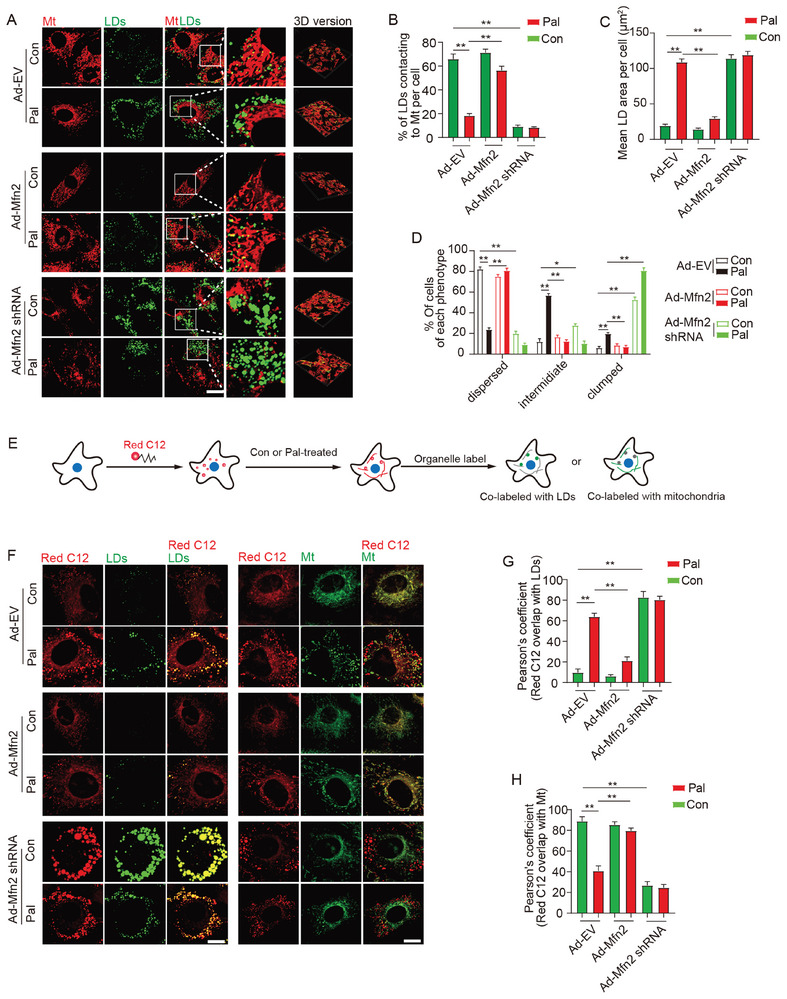
Mfn2 facilitated MLC and FAs transfer from LDs to mitochondria. A) Control‐ or Pal‐ treated primary cardiomyocytes were transfected with adenovirus expressing Mfn2 (Ad‐Mfn2) or Mfn2 shRNA (Ad‐Mfn2 shRNA), then LDs were labeled with Bodipy 493/503 (green) and mitochondria was labeled with Mito‐tracker Red (red). Original magnification ×600. B) Percentage of LDs with direct contact to mitochondria was measured (Mean± SEM, n = 5 independent experiments, 30 cells were quantified per group, **P<0.01). C) Mean LD area per cell (Mean± SEM, n = 5 independent experiments, 30 cells were quantified per group, **P<0.01). D) Percentage of cells treated as in A and presenting different degrees of LD dispersion (Mean± SEM, n = 5 independent experiments, 15 images were quantified per group, **P<0.01). E) Schematic illustration of FAs tracking assay. F) FAs were chased with Bodipy 558/568 Red C12 and imaged to determine the subcellular localization of the FAs (red). LDs and mitochondria were also labeled (green). Original magnification ×600. G and H) Relative localization of Red C12‐labeled FAs with LDs or mitochondria was quantified by Pearson's coefficient analysis (Mean± SEM, n = 5 independent experiments, 30 cells were quantified per group, **P<0.01).

To further elucidate the role of Mfn2 in the formation of MLC, we manipulated Mfn2 expression through adenovirus transfection, either overexpressing or knocking down Mfn2. Mfn2 overexpression significantly reduced the average LD area and reinstated MLC, leading to dispersed LDs in Pal‐treated cardiomyocytes (**Figure** **2**A–D). Conversely, Mfn2 knockdown markedly diminished MLC in cardiomyocytes, both with and without Pal treatment, resulting in clumped LDs clustered on the cell periphery (Figure 2A–D). It is widely acknowledged that MLC provides the structural basis for the transfer of FAs from LDs to the mitochondria. To visually demonstrate the Mfn2‐mediated MLC in FA transfer, FAs (labeled in red with C12) were co‐labeled with LDs (green) or mitochondria (green) in cardiomyocytes (Figure 2E). As illustrated in Figure 2F–H, nearly all FAs were localized within the mitochondria of control cardiomyocytes (Con+Ad‐EV), with few FA signals detected in the LDs. Pal‐treated cardiomyocytes exhibited a significant loss of FAs in the mitochondria, accompanied by an increased localization of FAs in the LDs. As expected, *Mfn2* overexpression in Pal‐treated cardiomyocytes facilitated the redistribution of FAs from LDs to mitochondria, while *Mfn2* knockdown hindered the transfer of FA signals from LDs to mitochondria (Figure 2E–H). A Red C12 pulse‐chase assay was also employed to further investigate the trafficking of FAs from LDs to mitochondria in the presence or absence of Mfn2. Cardiomyocytes were treated with Pal to induce LD formation, and FAs were labeled overnight with a trace amount of Red C12. The morphological change of Red C12 from red spots to red lines indicated the utilization of LD‐stored FAs by the mitochondria. As shown in Figure S3 (Supporting Information), most of the C12 FAs accumulated in neutral lipids within LDs after overnight Pal treatment. Subsequently, cells were placed in a nutrient‐depleted HBSS medium to induce FA utilization (Figure S3A, Supporting Information). Interestingly, cardiomyocytes transfected with Ad‐*Mfn2* exhibited a more evident transport of FAs from LDs to mitochondria throughout the pulse‐chase detection periods compared to cells transfected with Ad‐EV. Conversely, cells lacking Mfn2 showed a significant deficiency in FA utilization, with almost no FAs trafficking from LDs to mitochondria during the pulse‐chase assay (Figure S3B, Supporting Information). Moreover, mitochondrial respiration levels due to exogenous Pal were assessed by Seahorse extracellular flux analyzer to reflect mitochondrial FA oxidation. The data revealed that Mfn2 elevated maximal mitochondrial respiration due to the utilization of exogenous Pal, indicating enhanced mitochondrial lipid oxidation in Mfn2‐overexpressed cells (Figure S4A–C, Supporting Information). In summary, these findings suggest that Mfn2 promotes MLC and facilitates the transfer of FAs from LDs to the mitochondria for oxidation.

Given the well‐established role of Mfn2 in mitochondrial fusion, we conducted further investigations to determine whether Mfn2 promotes LD degradation by facilitating mitochondrial fusion. As anticipated, Mfn2 overexpression significantly enhanced mitochondrial fusion in Pal‐treated cardiomyocytes (Figure S5A–D, Supporting Information). Considering that Mfn1, Mfn2, and Opa1 participate in mitochondrial fusion, we attempted to restore mitochondrial fusion in Pal‐treated cardiomyocytes using adenoviruses encoding Mfn1 or Opa1 (Ad‐Mfn1 or Ad‐Opa1). As depicted in Figure S5E–H (Supporting Information), Ad‐Mfn1 or Ad‐Opa1 exhibited a notable effect in promoting mitochondrial fusion in Pal‐treated cardiomyocytes. However, neither Ad‐*Opa1* nor Ad‐*Mfn1* prevented LD accumulation (Figure S5G–J, Supporting Information). These findings suggest that the mechanism through which Mfn2 reduces LD accumulation may be independent of mitochondrial fusion.

### 2.3 Mitochondrion‐Localized Mfn2 Interacts with Hsc70 at The MLC

**Figure 3:**
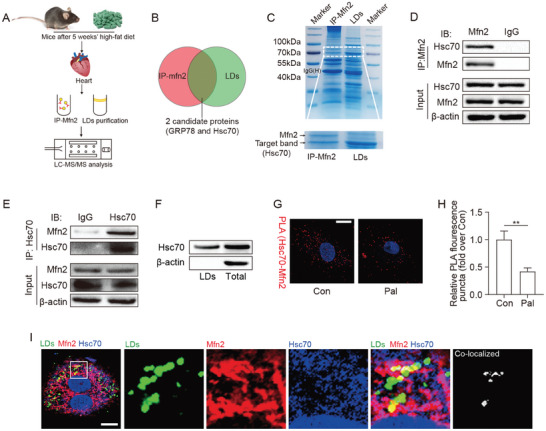
Mfn2 interacts with Hsc70 at MLC site. A and B) Schematic representation of the experimental protocols. C) Coomassie blue staining images for Mfn2‐binding proteins and LD proteins, band of Mfn2 and target proteins are labeled by arrow head. D and E) IP assay using different antibody to determine the binding of Mfn2 and Hsc70. F) Hsc70 expression in LDs were detected (n = 4 mice in each group). G and H) Representative pictures and quantitative analysis from proximity ligation assay (PLA) experiments in cardiomyocytes. PLA signals are visible as red dots. I) Fluorescence images show that Mfn2 (red) and Hsc70 (blue) colocalized on LD (green) surface, original magnification ×600, n = 5 independent experiments.

To delve into the mechanism by which Mfn2 regulates the formation of MLC, we conducted LD isolation and co‐immunoprecipitation (co‐IP) assays to identify LD proteins and proteins interacting with Mfn2 in the hearts of mice (**Figure** **3**A). LDs were successfully purified from the hearts of mice fed HFD for 5 weeks, devoid of other organelles such as mitochondria, ER, and cytosol (Figure S6A,B, Supporting Information). Subsequently, the samples underwent liquid chromatography‐tandem mass spectrometry analysis. The top 15 Mfn2‐binding proteins and the top 15 LD proteins are listed in Tables S2 and S3 (Supporting Information). Two candidates, Hsc70 and ER chaperone BiP (GRP78), were selected based on the intersection of Mfn2‐binding proteins and LD proteins (Figure 3B; Tables S2 and S3, Supporting Information). IP experiments revealed that Mfn2 interacted with Hsc70 but not GRP78 (Figure 3C–E; Figure S6C–D, Supporting Information). Western blotting confirmed the high levels of Hsc70 in LDs (Figure 3F). A duo‐link proximity ligation assay (PLA) facilitated the detection, visualization, and quantification of protein interaction within <40 nm distance as fluorescent dots. As depicted in Figure 3G–H, in situ PLA showed a noticeable signal for Mfn2‐Hsc70 proximity (red dots), which was further reduced after Pal treatment for 24 h. Immunofluorescence analysis indicated that Hsc70 (Figure 3I, blue) was enriched around the LD signal (green). Mfn2 (red) and Hsc70 (blue) co‐localized in a bead‐like structure (shown as white) around the LDs (green) (Figure 3I). High Hsc70 levels were also detected in the MLC region (Figure S7, Supporting Information).

### 2.4 Mitochondrion‐Localized Mfn2 Promotes MLC FA Transfer in An Hsc70‐Dependent Manner

**Figure 4:**
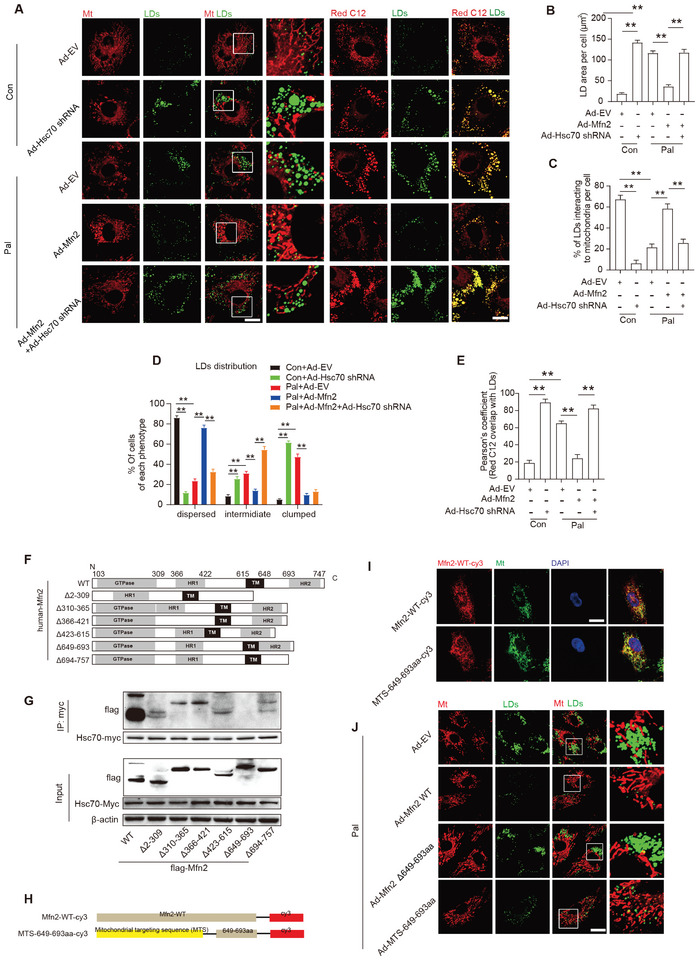
Mitochondrion‐localized Mfn2 promoted MLC FAs transfer in a Hsc70‐dependent manner. A) Control‐ or Pal‐ treated primary cardiomyocytes were transfected with control adenovirus (Ad‐EV) or adenovirus expressing Mfn2 (Ad‐Mfn2) or Hsc‐70 shRNA (Ad‐Hsc70 shRNA), then LDs were labeled with Bodipy 493/503 (green), mitochondria was labeled with Mito‐tracker Red (red) and FAs were labeled with red C12 (red) respectively. Original magnification ×600. B) Mean LDs area per cell (Mean± SEM, n = 5 independent experiments, 30 cells were quantified per group, **P<0.01). C) Percentage of LDs with direct contact to mitochondria in cells treated as in A (Mean± SEM, n = 5 independent experiments, 30 cells were quantified per group, **P<0.01). D) Percentage of cells treated as in A and presenting different degrees of LD dispersion (Mean± SEM, n = 5 independent experiments, 15 images were quantified per group, **P<0.01). E) Relative localization of Red C12 with LDs was measured in cells treated as in A (Mean± SEM, n = 5 independent experiments, 30 cells were quantified per group, **P<0.01). F) Schematic diagrams of Mfn2 structure and its truncated forms. G) 293T cell were transfected with wild‐type Mfn2 and its truncated mutant and applied to immunoprecipitation assay. H) Schematic diagrams showing recombinant sequence encoding cy3 labeled wild‐type Mfn2 and mitochondrial‐targeted Mfn2 649–693aa. I) Mitochondrial localization of Mfn2‐WT‐cy3 and MTS‐649‐693aa‐cy3 were indicated by fluorescent images. J) Palmitate‐treated primary cardiomyocytes were transfected with adenovirus expressing wild‐type Mfn2 (Ad‐Mfn2 WT) or 649–693aa deletion mutant Mfn2 (Ad‐Mfn2 Δ649‐693) or MTS‐649‐693aa, then LDs were labeled with Bodipy 493/503 (green) and mitochondria was labeled with Mito‐tracker Red (red).

To elucidate the role of Hsc70 in Mfn2‐mediated MLC formation and lipid metabolism, we knocked down Hsc70 in cardiomyocytes through adenovirus (Ad‐Hsc70 short hairpin RNA [shRNA]) transfection. Hsc70 knockdown resulted in increased LD content and disrupted MLC in control cells (**Figure** **4**A–C). Quantification of LD distribution revealed that the percentage of cells with dispersed LDs was significantly lower in Hsc70‐deficient cells (Figure 4A,D). Moreover, the transport of FAs away from the LDs was hindered by Hsc70 knockdown (Figure 4A,E). Additionally, Hsc70 knockdown inhibited the promoting effects of Mfn2 overexpression on MLC formation and FA transport, while counteracting the inhibitory effects of Mfn2 overexpression on LD content and dispersion (Figure 4A–E). Furthermore, maximal respiration due to the utilization of exogenous FAs in Ad‐Mfn2‐transfected cardiomyocytes was also inhibited by Hsc70 knockdown (Figure S9, Supporting Information). These data indicate that Hsc70 knockdown significantly impedes the effects of Mfn2 on MLC formation, FA transfer, and oxidation.

Several conserved functional domains in Mfn2 have been identified, including the GTPase, coiled‐coil (HR1), TM, and second coiled‐coil (HR2) domains.^[^ ^20^ ^]^ To pinpoint the specific domain of Mfn2 responsible for interacting with Hsc70, we generated six deletion mutations of Flag‐Mfn2 based on its established domains (Figure 4F). Co‐IP analysis demonstrated that the deletion of amino acids 649–693 remarkably abolished the Mfn2/Hsc70 interaction (Figure 4G). The 649–693 aa region was not a conserved domain shared by both Mfn1 and Mfn2 (Figure S8, Supporting Information). Despite Mfn2's predominant intracellular localization in the mitochondria, it was also found in other cellular components, such as the ER, cytoplasm, and lysosomes, albeit to a lesser extent. To further ascertain the crucial role of mitochondrial‐localized Mfn2 in MLC and LD metabolism, mitochondrial‐targeted Mfn2 649–693 aa constructs were generated by fusing the Mfn2 649–693 aa with the mitochondrial targeting sequence (MTS) of Fundc1. As shown in Figure 4I, compared with WT Mfn2, MTS‐649–693 aa exhibited a more pronounced co‐localization with mitochondria. The MTS‐649–693 aa‐cy3 signal was almost exclusively localized in the mitochondria. Importantly, the truncated mutant of Mfn2 lacking 649–693 aa showed a significant effect in promoting mitochondrial fusion but failed to restore MLC formation in Pal‐treated cardiomyocytes (Figure 4J). In contrast, adenovirus encoding MTS‐649–693 aa transfection restored MLC and prevented LD accumulation but was unsuccessful in promoting mitochondrial fusion (Figure 4J), mitochondria‐sarcoplasmic reticulum tethering (Figure S10A, Supporting Information), and mitophagy (Figure S10B, Supporting Information). These results suggest that mitochondrion‐localized Mfn2 interacts with Hsc70 via its 649–692 aa domain, facilitating the formation of MLC and preventing lipid overload‐induced LD accumulation.

### 2.5 Cardiac‐Specific Knockout of Mfn2 in Mice Reduces MLC and Increases Intra‐Myocardial Lipid Accumulation

To investigate the impact of Mfn2 on MLC in vivo, we utilized cardiac‐specific Mfn2 knock‐out (Mfn2^CKO^) mice obtained through the crossbreeding of Mfn2^fl/fl^ with *αMyHC*‐*Mer*‐*Cre*‐*Mer* mice. Specific Mfn2 deletion was observed in the heart tissue of Mfn2^CKO^ mice, with no detectable knockout in other tissues, such as skeletal muscle and liver (Figure S11A, Supporting Information). The expression of genes associated with lipolysis (i.e., ATGL, CPT‐1b, and CPT‐2) significantly decreased in Mfn2^CKO^ hearts (Figure S11B, Supporting Information).

**Figure 5:**
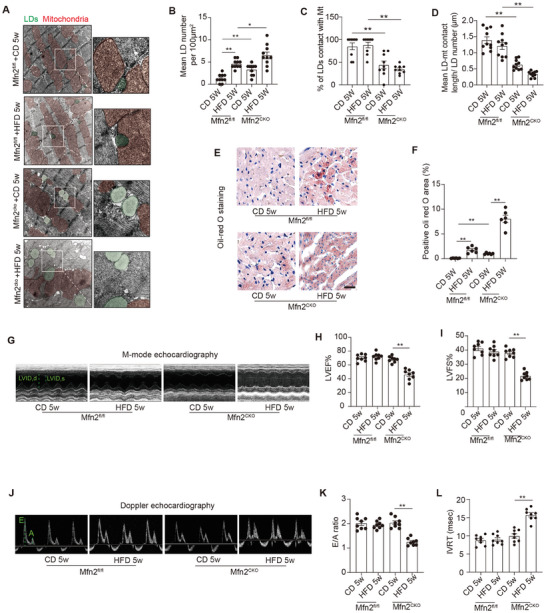
Cardiac‐specific Mfn2 knock‐out mice displayed reduced MLC and increased intra‐myocardial lipid accumulation. A) Representative electron microscopy images of mitochondria and LDs from hearts of Mfn2^fl/fl^ and Mfn2^CKO^ mice after 5 weeks’ CD or HFD feeding. LDs are labeled as green while mitochondria are labeled as red. Scale bars, 1 µm. B) Intra‐myocardial LD number were calculated to assess lipid accumulation in myocardium (Mean± SEM, n = 10 images from 8 mice each group, **P<0.01). C) Percentage of LDs contact with mitochondria (Mean± SEM, n = 10 images from 8 mice each group, **P<0.01). D) Mean LD‐mitochondria contact length were measured to reflect LD‐mitochondria tethering in myocardium. (Mean± SEM, n = 10 images from 8 mice each group, **P<0.01). E) Oil‐red O staining showing intra‐myocardial lipid content, oil‐red O positive area are shown as red, scale bar = 25 µm. F) Quantitative analysis of positive Oil‐red O area in each group (Mean± SEM, n = 6 mice each group, **P<0.01). G–I) Echocardiographic assessment were performed on Mfn2^fl/fl^ and Mfn2^CKO^ mice after 5 weeks’ CD or HFD feeding. n = 8 mice per group. G) Representative images of M‐mode echocardiography. H and I) left ventricle ejection fraction (LVEF) and left ventricle fractional shortening (LVFS). J) Representative Doppler flow measurement of mitral inflow. K) E/A ratio. L) isovolumic relaxation time (IVRT). (Mean± SEM, **P<0.01). CD, chow diet; HFD, high‐fat diet; LD, lipid droplet.

Building upon our prior findings that Mfn2 expression increased following 5 weeks of HFD feeding, both Mfn2^CKO^ mice and their control littermates (Mfn2^fl/fl^) were subjected to either a standard CD or HFD for 5 weeks. Under both CD and HFD conditions, the hearts of Mfn2^CKO^ mice exhibited markedly reduced MLC, as evidenced by a decreased percentage of LDs in contact with mitochondria and shorter MLC length (**Figure** **5**A,C,D). Additionally, intra‐myocardial LD number and lipid accumulation were higher in Mfn2^CKO^ mice compared to Mfn2^fl/fl^ mice under both CD and HFD conditions (Figure 5A,B,E,F). Notably, Mfn2^CKO^ mice displayed pronounced cardiac systolic and diastolic dysfunction after 5 weeks of HFD feeding (Figure 5G–L).

Intramyocardial lipid content was further scrutinized through lipidomics (Figure S12, Supporting Information). TAGs constitute the primary form of lipid storage, while diglycerides (DAG) and ceramides are^[^ ^5^ ^]^ recognized as prominent contributors to lipotoxicity.^[^ ^5^ ^]^ Accordingly, we measured TAG, DAG, cholesterol, and ceramides in mice. Mfn2 knockout resulted in elevated levels of nearly all TAG species in the hearts of mice fed a standard CD (Figure S12A,B, Supporting Information). HFD significantly increased intramyocardial levels of TAG, DAG, and ceramides in the hearts of control mice (Mfn2^fl/fl^) (Figure S12A–E, Supporting Information). Noteworthy, Mfn2 knockout exacerbated the accumulation of lipid species in the hearts of HFD‐fed mice, including TAG and C16:0 DAG (Figure S12A–E, Supporting Information), a lipid class implicated in oxidative stress and apoptosis.^[^ ^21^ ^]^

Furthermore, as depicted in Figure S13 (Supporting Information), Mfn2 ablation in mouse hearts elevated the levels of cardiolipin 36:3/36:4, 36:3/34:1, and 34:1/34:2, while reducing the level of cardiolipin 36:4/36:5. Collectively, these findings indicate that Mfn2 ablation hinders MLC formation and induces intra‐myocardial lipid accumulation, contributing to lipotoxicity and consequent cardiac dysfunction.

### 2.6 Cardiac‐Specific Overexpression of Mfn2 Prevents HFD‐Induced Suppression of MLC and Intra‐Myocardial Lipid Accumulation via Hsc70

We engineered cardiac‐specific Mfn2 transgenic (Mfn2^TG^) mice by crossing Rosa26‐CAG‐Loxp‐Stop‐Loxp‐Mfn2 knock‐in mice with α‐MHC‐Mer‐Cre‐Mer mice. Verification of cardiac‐specific Mfn2 overexpression in the hearts of Mfn2^TG^ mice was confirmed through western blotting (Figure S14A, Supporting Information). In contrast to control littermate mice (i.e., non‐transgenic [NTG] *Rosa26*‐*CAG*‐*Loxp*‐*Stop*‐*Loxp*‐*Mfn2* knock‐in mice), the hearts of Mfn2^TG^ mice exhibited a significantly enhanced lipolytic capacity, supported by increased gene expression of ATGL and CPT‐2 (Figure S14B, Supporting Information).

Based on our prior findings that 10 weeks of HFD feeding reduced Mfn2 expression and MLC formation, Mfn2^TG^ mice and their control littermates were subjected to either a standard CD or HFD for 10 weeks. Mfn2 overexpression effectively increased MLC formation in HFD‐fed mice (Figure S12A,D,E, Supporting Information). Furthermore, Mfn2 overexpression led to a reduction in intramyocardial LD numbers, mean LD diameters, and the quantification of oil‐red O‐positive areas in HFD‐fed mice (Figure S15A–F, Supporting Information). Echocardiography data indicated a significant improvement in cardiac diastolic functions in HFD‐fed mice with Mfn2 overexpression (Figure S15G–L, Supporting Information). These in vivo results collectively demonstrate that cardiac Mfn2 overexpression acts as a protective mechanism, preventing HFD‐induced MLC suppression and reducing intra‐myocardial lipid accumulation, ultimately safeguarding against HFD‐induced cardiac dysfunction.

**Figure 6:**
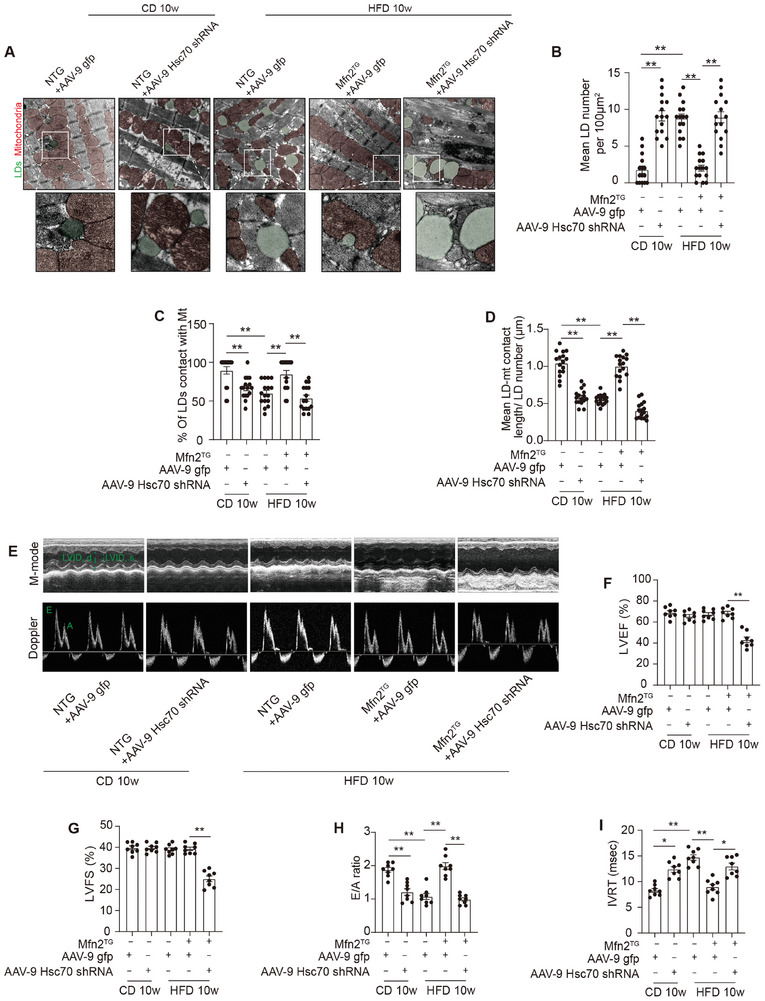
Hsc70 knock‐down induced mitochondria‐LDs disassociation and lipid accumulation in hearts of NTG and Mfn2^TG^ mice. A) Mice were intra‐myocardial injected with control adeno‐associated virus 9 (AAV‐9) or recombined AAV‐9 encoding Hsc70 shRNA and then subjected to10 weeks’ CD or HFD feeding. LDs are labeled as green while mitochondria are labeled as red. Scale bars, 1 µm. B) Intra‐myocardial LD number were calculated to assess lipid accumulation in myocardium (Mean± SEM, n = 15 images from 8 mice each group, **P<0.01). C) Percentage of LDs contact with mitochondria (Mean± SEM, n = 15 images from 8 mice each group, **P<0.01). D) Mean LD‐mitochondria contact length were measured to reflect LD‐mitochondria tethering in myocardium. (Mean± SEM, n = 15 images from 8 mice each group, **P<0.01). E–I) Echocardiographic assessment were performed on NTG and Mfn2^TG^ mice treated as indicated. n = 8 mice per group. E) Representative images of M‐mode echocardiography (above) and representative Doppler flow measurement of mitral inflow (below). F–I) left ventricle ejection fraction (LVEF), left ventricle fractional shortening (LVFS), E/A ratio and isovolumic relaxation time (IVRT). (Mean± SEM, **P<0.01). CD, chow diet; HFD, high‐fat diet; LD, lipid droplet.

The expression of Hsc70 in the hearts of HFD‐fed mice showed a slight increase (Figure S16A, Supporting Information). To further elucidate the role of Hsc70 in MLC and related lipid metabolism in vivo, we employed a knockdown approach using adeno‐associated virus 9 (AAV‐9) delivered through intramyocardial injections. AAV‐9 carrying Hsc70 shRNA effectively reduced Hsc70 expression in WT, NTG, and Mfn2^TG^ mice (Figure S16B–D, Supporting Information). Hsc70 knockdown hindered MLC in the hearts of control mice (**Figure** **6**A–D), with a significant increase in LD numbers, suggesting a pivotal role for Hsc70 in MLC and cardiac lipid metabolism (Figure 6A–B).

Furthermore, Hsc70 knockdown significantly impaired cardiac diastolic function in NTG mice, while systolic function remained unaffected (Figure 6E–I). Additionally, Hsc70 knockdown attenuated the promoting effects of Mfn2 overexpression on MLC formation and cardiac diastolic function, while counteracting the inhibitory effects of Mfn2 overexpression on myocardial LD accumulation (Figure 6A–I). These findings indicate that Hsc70 plays a crucial role in facilitating Mfn2‐mediated regulation of MLC and lipid metabolism.

### 2.7 Lipid Overload Promotes Acetylation of Mfn2 at The K243 Site and Its Degradation through The Ubiquitin‐Proteasome Pathway

**Figure 7:**
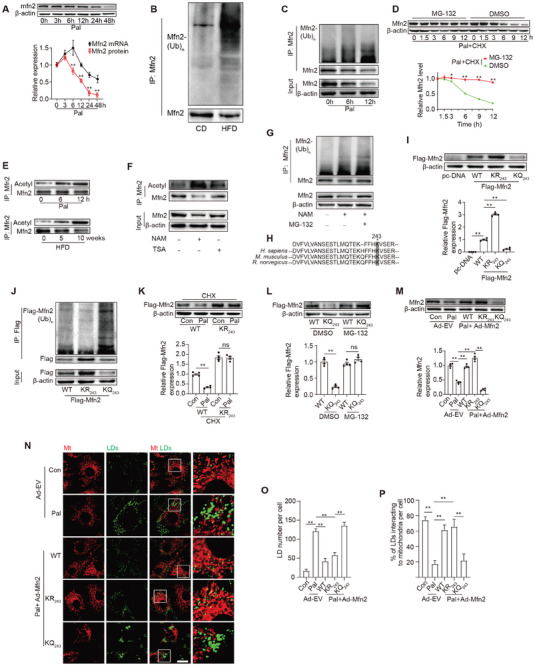
Lipid overload promoted Mfn2 acetylation at K243 site and further degradation via a ubiquitin‐proteasome pathway. A) Quantification of Mfn2 protein and mRNA level in cardiomyocytes at different time points of Pal treating (Mean± SEM, n = 4 independent experiments, **P<0.01). B,C) Mfn2 ubiquitylation were detected in heart of HFD‐fed mice and palmitate‐treated primary cardiomyocytes (Mean± SEM, n = 4 independent experiments, **P<0.01). D) Palmitate‐treated cardiomyocytes were incubated with CHX (10 µM) and MG‐132 (10 µM) for the indicated periods of time. Mfn2 level were analyzed by western‐blotting. E) Acetylation level of Mfn2 were determined in vivo and in vitro at different time points of HFD‐feeding or palmitate treating (n = 4 mice in each group in vivo and 4 independent experiments in vitro). F) Trichostatin A (inhibitor of inhibitor of histone deacetylase, TSA, 50 nM) or nicotinamide (inhibitor of Sirtuins family, NAM, 20 mM) were used to induce Mfn2 acetylation in cardiomyocytes, and Mfn2 acetylation level were determined (n = 4 independent experiments). G) Mfn2 protein and ubiquitylation level were determined in cardiomyocytes in the presence of NAM or MG‐132. H) Alignment of the Mfn2 protein sequences surrounding lysine 243 from mouse to human, conserved lysine residues are shaded. I) Mfn2 K243 mutation leads to different protein level of Flag‐Mfn2 (Mean± SEM, n = 4 independent experiments, **P<0.01). J) Mfn2 protein and Mfn2 ubiquitylation level were determined (Mean± SEM, n = 4 independent experiments, **P<0.01). K) Western blotting images and summarized data showing the degradation of Flag‐Mfn2 (wild type [WT]) or Flag‐Mfn2 KR243 mutation (KR243) with or without palmitate and Chlorhexidine (CHX, 10 µM) incubation. L) Western blotting images and summarized data showing the degradation of Flag‐Mfn2 (wild type [WT]) or Flag‐Mfn2 KQ243 mutation (KQ243) with or without MG‐132 (10 µM) incubation (Mean± SEM, n = 4 independent experiments, **P<0.01). M) Mfn2 expression were determined by Western‐blot in cardiomyocytes as indicated. N) LDs were labeled with Bodipy 493/503 and mitochondria was labeled with Mito‐tracker Red. Original magnification ×600. O) LDs number per cell (Mean± SEM, n = 5 independent experiments, 30 cells were quantified per group, **P<0.01). P) Percent of LDs interacting to mitochondria per cell (Mean± SEM, n = 5 independent experiments, 30 cells were quantified per group, **P<0.01).

Further exploration of the mechanisms underlying lipid overload‐induced Mfn2 downregulation revealed alterations in protein and mRNA levels in Pal‐treated cardiomyocytes at different time points. **Figure** **7**A illustrates that both Mfn2 protein and mRNA levels increased 3 h after Pal exposure. Notably, after 12 h Pal treatment, the Mfn2 protein level significantly decreased, while the *Mfn2* mRNA level remained relatively unchanged (compared to Pal 0 h). Following 24 or 48 h Pal treatment, the Mfn2 protein level markedly decreased compared to the mRNA level. In vivo results also demonstrated a significant reduction in Mfn2 protein levels in the hearts of 10‐week HFD‐fed mice, with a slight decrease in mRNA levels (Figure 1L; Figure S17, Supporting Information). These findings indicate that post‐transcriptional mechanisms impede Mfn2 protein levels in response to lipid overload.

The ubiquitin‐proteasome pathway, a common route for protein degradation, was investigated, revealing a significant increase in Mfn2 ubiquitination in the hearts of HFD‐fed mice and Pal‐treated cardiomyocytes (Figure 7B,C). Consequently, cells were treated with the ubiquitin‐proteasome pathway inhibitor MG‐132. To mitigate the potential effects of Pal on protein synthesis, cycloheximide (CHX) was employed to block protein synthesis. As depicted in Figure 7D, Pal treatment in CHX‐treated cells led to a time‐dependent decrease in Mfn2 protein levels, which was reversed by the proteasome inhibitor MG‐132. These data suggest that Pal treatment induces Mfn2 degradation via the ubiquitin‐proteasome pathway.

Lipid overload induces an intracellular environment that promotes protein acetylation.^[^ ^22^ ^]^ Consequently, we investigated the correlation between Mfn2 ubiquitination and acetylation. As anticipated, lipid overload led to a time‐dependent increase in Mfn2 acetylation in both primary cardiomyocytes and mouse hearts (Figure 7E). Nicotinamide (NAM), an inhibitor of the sirtuin deacetylase family (but not TSA, a histone deacetylase inhibitor), elevated Mfn2 acetylation levels (Figure 7F). Furthermore, NAM increased the ubiquitination of Mfn2 and reduced Mfn2 protein levels, effects that were mitigated by MG‐132 (Figure 7G).

A previous study identified K243 as a potential site for Mfn2 acetylation using tandem affinity purification‐coupled mass analysis.^[^ ^23^ ^]^ Our results revealed a high conservation of the neighboring amino acids ∼K243 (Figure 7H). Subsequently, we generated a point mutation at the K243 site of Mfn2 to mimic the nonacetylated (K243R) or acetylated (K243Q) state. The acetylation‐deficient K243R mutant decreased Mfn2 ubiquitylation and increased Mfn2 protein levels, while the acetylation‐mimicked K243Q mutant increased Mfn2 ubiquitylation and reduced Mfn2 protein levels (Figure 7I–J). Pal incubation decreased Mfn2 protein levels in WT‐ but not in K243R‐*Mfn2* overexpressing cardiomyocytes (Figure 7K). In contrast, the inhibitory effect of the K243Q mutation on Mfn2 was reversed by MG‐132 treatment (Figure 7L). These findings strongly indicate that acetylation at K243 of Mfn2 is crucial for protein stability. Furthermore, overexpression of the WT or K243R mutant of *Mfn2*, but not the K243Q mutant, reduced LD content and promoted MLC in Pal‐treated cardiomyocytes (Figure 7M–P). These data suggest that lipid overload induces Mfn2 acetylation at K243, facilitating its degradation via the ubiquitin‐proteasome pathway.

### 2.8 Mfn2‐Hsc70 Mediates MLC and Lipid Accumulation in Pal‐Treated Human Induced Pluripotent Stem Cell‐Derived Cardiomyocytes (hiPSC‐CMs) and Cardiac Specimens Collected from Patients with Obesity

To validate the translational potential of our experimental findings, hiPSC‐CMs were employed. The cardiac ontogeny of hiPSC‐CMs was confirmed by analyzing cardiac cell‐specific transcriptional, structural, and functional markers (Figure S15A,B, Supporting Information). Pal treatment significantly decreased Mfn2 levels and disrupted MLC formation in hiPSC‐CMs, concomitant with a substantial accumulation of LDs (Figure S15C,F,G, Supporting Information). Additionally, Pal treatment hindered the transfer of Fas away from LDs in hiPSC‐CMs (Figure S15D,H, Supporting Information). *Mfn2* overexpression restored MLC formation, redirected FAs from LDs to the mitochondria, and prevented LD accumulation in Pal‐treated hiPSC‐CMs (Figure S15C–H, Supporting Information). Conversely, *Hsc70* knockdown induced noticeable MLC disruption, resulting in abnormal lipid accumulation and deposition of FAs in the LDs of hiPSC‐CMs (Figure S15I–N, Supporting Information).

**Figure 8:**
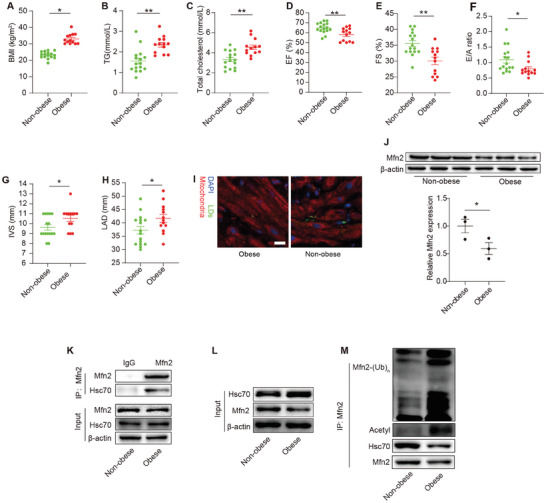
Decreased Mfn2 expression and reduced MLC were observed in cardiac specimens from obese patients. A–H) BMI, TG, total cholesterol, EF, FS, E/A ratio, IVS and LAD are compared between obese patients (n = 13) and non‐obese individuals (n = 16) (Mean± SEM, *P<0.05, **P<0.01). I) mitochondria and LDs were co‐staining in the frozen human atrial tissue from obese or non‐obese individuals. n = 3 per group. J) Mfn2 protein expression in atrial tissue from non‐obese or obese individuals were determined by western‐blot, n = 3 per group. K) IP assay were performed in human atrial tissue to detect the binding of Mfn2 and Hsc70. L and M) Mfn2‐Hsc70 interaction, Mfn2 acetylation level and Mfn2 ubiquitylation level were determined in human atrial tissue from obese and non‐obese individuals.

Right atrial specimens were collected from individuals with obesity (BMI ≥30 kg m^−2^) and age‐matched counterparts in the clinic. Notably, obese individuals exhibited elevated serum TGs (i.e., triglycerides) and cholesterol levels, despite a higher rate of statin use (**Figure** **8**A–C, Table S4, Supporting Information). Echocardiography data indicated impaired cardiac systolic and diastolic function in patients with obesity compared to those without obesity (Figure 8D–F, Table S4, Supporting Information). Patients with obesity also displayed higher left atrial diameters and increased interventricular septum thicknesses (Figure 8G–H, Table S4, Supporting Information). In Figure 8I, it is evident that these patients had more intra‐myocardial LDs, most of which were not in contact with the mitochondria. Moreover, Mfn2 levels were lower in the myocardium of patients with obesity than in controls (Figure 8J). The co‐IP assay in human myocardium revealed decreased binding of Mfn2 with Hsc70 in the hearts of patients with obesity (Figure 8K), which was associated with increased Mfn2 acetylation and ubiquitylation (Figure 8L,M).

## 3 Discussion

In this study, we have discovered, for the first time, that mitochondrion‐localized Mfn2 binds to LD‐localized Hsc70 to mediate the interaction between mitochondria and LDs, thereby facilitating the transfer of Fas from LDs to mitochondria for oxidation. In the hearts of normal or short‐term HFD‐fed mice, the preserved level of Mfn2 contributes to a tight MLC and adaptive lipid utilization. As lipid overload persists, Mfn2 is notably acetylated at the K243 site, leading to its degradation through a ubiquitin‐proteasome pathway. The degradation of Mfn2 in cardiomyocytes disrupts MLC, hindering the transfer of FAs to mitochondria, resulting in intramyocardial lipid accumulation and maladaptive lipotoxicity (see Graphical abstract).

In cardiomyocytes, MLC appears to form a specialized synapse that tightly regulates the mobilization and oxidation of FAs for ATP production.^[^ ^24^ ^]^ However, the molecular machinery regulating this contact is largely unknown. In this study, we have identified, for the first time, that mitochondrion‐localized Mfn2 binds to LD‐localized Hsc70 to mediate MLC, facilitating the transfer of FAs from LDs to mitochondria for oxidation. First, Mfn2 physically interacts with Hsc70 at the MLC site, confirmed by co‐IP assays and further immunofluorescence detection. Second, the ablation of Mfn2 or Hsc70 results in the disassociation between mitochondria and LDs in both mouse hearts and cultured cardiomyocytes, leading to lipid accumulation and severe HFD‐induced cardiomyopathy. It is noteworthy that no previous study has reported cardiac lipid accumulation and lipotoxicity in the hearts of Mfn2 cardiac‐specific knockout (CKO) mice. However, a recent study indicated that Mfn2 deficiency reduced the capacity for cardiac FA oxidation,^[^ ^25^ ^]^ consistent with our experimental results. Furthermore, Song et al. reported that Mfn2 deficiency induced severe dilated cardiomyopathy and progressive heart failure in 30‐week‐old normal‐feeding mice.^[^ ^26^ ^]^ Unfortunately, lipid accumulation was not determined in 30‐week‐old Mfn2^CKO^ mice. It cannot be expected that LD accumulation and impairment of FA oxidation capacity induced by prolonged Mfn2 deficiency are involved in the development of progressive heart failure. In contrast to binding with Hsc70 in cardiomyocytes, Mfn2 interacts with Plin1 (an LD coat protein) in brown adipose tissue (BAT) to function as a tethering complex.^[^ ^27^ ^]^ Notably, unlike its abundant levels in BAT, Plin1 is undetectable in hearts,^[^ ^28^ ^]^ indicating that the mechanisms by which Mfn2 regulates MLC are inconsistent among different organelles. Furthermore, Mfn2 overexpression promoted the distribution of FAs from LDs to mitochondria in lipid‐overloaded cardiomyocytes, whereas Mfn2 or Hsc70 knockdown led to FA accumulation in LDs and inhibited mitochondrial FA oxidation. These results are consistent with previous studies suggesting that MLC might provide a structural basis for FAs transfer and coupling lipolytic processes with β‐oxidation.^[^ ^29^, ^30^ ^]^ Interestingly, a recent study examining LD‐associated mitochondria (i.e., peri‐droplet mitochondria) in BAT suggests an opposing model, in which LD‐associated mitochondria exhibited reduced β‐oxidation, indicating that MLC enables cells to perform antagonistic metabolic processes.^[^ ^31^ ^]^ The contact between LDs and mitochondria could play roles in both lipogenesis and lipolysis in different tissues, with adipose tissue primarily functioning in lipid storage while the heart utilizes massive lipids for ATP synthesis.

Mfn1 and Mfn2 are situated on the outer membrane of the mitochondria and are implicated in outer membrane fusion, while inner membrane fusion is facilitated by Opa1. It has been reported that Mfn1 and Mfn2 share structural similarity, allowing Mfn1 to partially compensate for some functions of Mfn2 but not all.^[^ ^16^, ^32^ ^]^ Apart from its role in mitochondrial fusion, Mfn2 seems to have non‐canonical functions in membrane contact, such as tethering the ER to mitochondria and facilitating autophagosome‐lysosome fusion. Previous research from our team demonstrated that Mfn2 safeguards against diabetic cardiomyopathy by promoting mitochondrial fusion.^[^ ^19^ ^]^ However, this study reveals that Mfn2's role in MLC is independent of its promotion of mitochondrial fusion and cannot be substituted by Mfn1. In contrast to our findings, Rambold et al. proposed that mitochondrial fusion ensures the optimal distribution of FAs throughout the mitochondria for maximal β‐oxidation in starved cells.^[^ ^33^ ^]^ This discrepancy may be attributed to the unaffected MLC and Mfn2 levels in the starved cells. Mfn1‐ or Opa1‐mediated mitochondrial fusion seems to contribute to the distribution and oxidation of FAs, provided that MLC is not hindered. Our fragment analysis indicates that the function of Mfn2 in MLC relies on its 649–693 aa domain, which is not a conserved domain residing within Mfn1.^[^ ^20^ ^]^ Furthermore, the 649–693 aa domain of Mfn2 is not involved in mitochondrial fusion.^[^ ^20^ ^]^ In line with our results, a previous study demonstrated that the absence of Mfn1 or Opa1 did not impact the overall release of FAs from LDs.^[^ ^33^ ^]^

Apart from its role as a mitochondrial‐located protein, Mfn2 is also found in other subcellular compartments, including the ER, cytoplasm, and lysosomes. It has been reported that ER‐localized Mfn2 can regulate mitochondrial activity. In this study, we generated mitochondrial‐targeted Mfn2 fragments and provided compelling evidence that the Hsc70‐binding domain of Mfn2, residing in mitochondria alone, could induce the phenotype observed with WT Mfn2 overexpression. These findings suggest that mitochondrial‐localized Mfn2, and not Mfn2 in other subcellular compartments, plays a crucial role in the formation of MLC and lipolysis. In summary, our study offers novel insights into the function of Mfn2 in MLC and lipid metabolism.

Hsc70 has been implicated as a molecular chaperone, playing a role in protein translocation, folding, and sorting within cells.^[^ ^34^ ^]^ In chaperone‐mediated autophagy, Hsc70 recognizes the pentapeptide motif of proteins and transports them to the lysosome surface, where they bind to lysosome‐related membrane proteins.^[^ ^35^, ^36^ ^]^ Additionally, Hsc70 has been reported to directly interact with lipids and may be involved in membrane protein folding and the translocation of polypeptides.^[^ ^37^ ^]^ Previous studies have documented the presence of Hsc70 on the surface of LDs, and it persists at the LDs surface when chaperone‐mediated autophagy degradation is blocked.^[^ ^38^, ^39^, ^40^, ^41^ ^]^ Furthermore, Hsc70 has been described to interact with the LD surface protein perilipin 3, participating in the targeted removal of perilipins from the LD surface. This removal facilitates the access of lipolysis enzymes to the stored TAGs.^[^ ^41^ ^]^

Combined with the results of the present study, it becomes evident that TAGs, broken down by lipolysis enzymes, are subsequently transferred to mitochondria via MLC for complete β‐oxidation. The entire process of lipid degradation occurring at MLC sites is likely to be intricate. Nevertheless, our study takes a further step by providing a comprehensive understanding of the role of Hsc70 in LD metabolism. Hsc70 exerts dual regulatory functions by facilitating the degradation of perilipins and contributing to the formation of MLC.

Moreover, we investigated how lipid overload leads to the disconnection of mitochondria and LDs, contributing to maladaptive lipotoxicity in the hearts of individuals with obesity. The cumulative lipid load promoted the acetylation of Mfn2 at the K243 site, subsequently leading to its degradation through the ubiquitin‐proteasome pathway. While Mfn2 phosphorylation, acetylation, and ubiquitination have been reported previously,^[^ ^42^, ^43^, ^44^ ^]^ our study is the first to unveil that the acetylation status of Mfn2 controls its stability. A prior study demonstrated that JNK‐triggered Mfn2 phosphorylation induces Mfn2 degradation through ubiquitin ligase under doxorubicin treatment.^[^ ^45^ ^]^ In contrast, our results highlight a novel post‐transcriptional modification (PTM) of Mfn2 in response to lipid overload, suggesting that cells may adopt different PTMs in response to various cellular stresses. Moreover, lipid overload broadly influences protein acetylation and function.^[^ ^22^, ^46^, ^47^ ^]^ We observed that the protein level of Mfn2 was modulated differently by transcription and PTM at different stages of lipid overload. In the early phase of lipid overload, it appears that the increased transcriptional level of Mfn2 exceeds its PTM regulation, resulting in a slight increase in its protein level. However, with prolonged lipid overload, cumulative acetylation gradually dominates the regulation of Mfn2, leading to the disconnection of mitochondria and LDs and aberrant lipid accumulation. These findings may partly elucidate why short‐term superabundant lipid supply does not adversely affect cardiac function in healthy individuals, whereas long‐term lipid overload causes significant maladaptive lipotoxicity. Therefore, we propose a novel lipid overload‐induced Mfn2 PTM pathway that plays a key role in the transition from adaptive lipid utilization to maladaptive lipotoxicity.

A previous study reported that patients with obesity exhibit higher intramyocardial lipid staining.^[^ ^48^ ^]^ Furthermore, a significant reduction in glutamate and FA‐induced respiration capacity, along with an elevated intramyocardial TAG content, has been documented in the atrial tissue of patients with type 2 diabetes mellitus.^[^ ^8^ ^]^ In the current study, we observed an increased number of intramyocardial LDs in patients with obesity, most of which were not connected to the mitochondria, indicating a decoupling between lipid storage and lipid oxidation in the hearts of these patients. Additionally, we observed the inhibition of MLC and a decrease in Mfn2 levels in Pal‐treated hiPSC‐CMs and cardiac tissue from patients with obesity. Mfn2 overexpression restored MLC formation, facilitated the redistribution of FAs from LDs to mitochondria, and prevented LDs accumulation in Pal‐treated hiPSC‐CMs. These findings further support the concept that restoring MLC is a viable approach for developing new therapeutic strategies to address myocardial lipotoxicity.

Our study has several limitations. First, besides its localization on mitochondria, Mfn2 is also present in the ER,^[^ ^49^ ^]^ which is reported to interact with LDs and participate in LD formation.^[^ ^50^ ^]^ While mitochondria play a dominant role in cardiac LD metabolism, the contribution of Mfn2 in LD‐ER interaction cannot be excluded. Further studies are necessary to elucidate this aspect. issue. Second, due to ethical constraints, our exploration was limited to atrial tissues of human hearts, potentially not fully representing overall cardiac conditions. Third, in this study, Red C12 was employed as a fluorescence probe to track the FAs transport and distribution in the live cells. However, Red C12 can be used as a synthetic precursor to a wide variety of fluorescent phospholipids, indicating that Red C12 can also label phospholipid. Phospholipids mainly form components of biological membranes. In this study, we found that most Red C12 was co‐labeled within either LDs or mitochondria in the cardiomyocytes, suggesting that the amount of Red C12‐labeled phospholipids (membrane components) is much less than that of FAs. Therefore, the majority of the Red C12 staining can be a reflection of FAs transport. Future development and utilization of FAs‐specific labeling probes may help to address this issue. Last, although we identified the Mfn2‐Hsc70 complex as a major mediator for MLC, the possibility of another form of membrane contact between mitochondria and LDs cannot be ruled out. Further investigations are warranted to clarify the comprehensive structural and functional contacts between LDs and mitochondria.

In conclusion, our study demonstrated that Mfn2 binds to LD‐localized Hsc70 to mediate MLC, facilitating the transfer of FAs from LDs to mitochondria in cardiomyocytes. Lipid overload triggers acetylation of Mfn2 at the K243 site, promoting its degradation through the ubiquitin‐proteasome pathway. Consequently, reduced Mfn2 levels diminish MLC formation and hinder FA transfer, resulting in lipid accumulation and cardiac damage. This study, for the first time, unveils the molecular basis of MLC in the heart and investigates its role in myocardial lipotoxicity. Thus, manipulating MLC could represent a novel strategy for therapeutic interventions in lipid overload‐related cardiac dysfunctions.

## 4 Experimental Section

The detailed material and methods section can be found in the Supplemental Materials and Methods.

### Study Approval

The study protocol received approval from the Local Ethics Committee (IEC of the Institution for National Drug Clinical Trials, Tangdu Hospital, Fourth Military Medical University, China; #202103‐12). Prior to their participation, all participants were fully informed about the investigational nature of the study and provided written informed consent. Animal experiments were conducted in accordance with protocols approved by the Institutional Animal Care and Use Committee at the Fourth Military Medical University (Xi'an, China).

### Statistical Analysis

All values presented in this study were expressed as mean ± standard error (SEM). Differences between two groups were analyzed using Student's *t*‐test (two‐tailed), and multiple comparisons were assessed by ANOVA with a Bonferroni post hoc test using GraphPad Prism v8.0. Categorical variables were compared using the χ^2^ test. A value of *P*<0.05 was considered statistically significant.

## Conflict of Interest

The authors declare no conflict of interest.

## Author Contributions

L.H., D.T., and B.Q. contributed equally to this work. F.F. and Y.L. conceived and designed the study. L.H., D.T., B.Q., D.G., T.P., and Y.W. performed the animal experiments. L.H., Y.W., M.Z., P.C., J.G., and X.Z. carried out the cell experiments. L.H., W.C., F.F., and Y.L. analyzed the data. L.H. drafted the manuscript. F.F. and Y.L. revised and edited the manuscript. All authors have read and approved the final version of this manuscript.

## Supporting information

Supporting Information

## Data Availability

The data that support the findings of this study are available on request from the corresponding author. The data are not publicly available due to privacy or ethical restrictions.
