# Supplementary material for: Mfn2/Hsc70 Complex Mediates the Formation of Mitochondria‐Lipid Droplets Membrane Contact and Regulates Myocardial Lipid Metabolism
Source: Adv Sci (Weinh). 2024 Feb 4;11(14):2307749. doi: 10.1002/advs.202307749 (PMC11005711; doi:10.1002/advs.202307749)
Supplement: Supplementary file 1 — Supporting Information [file ADVS-11-2307749-s001.pdf]

ADVANCED SCIENCE
Open Access

Supporting Information

for *Adv. Sci.*, DOI 10.1002/advs.202307749

Mfn2/Hsc70 Complex Mediates the Formation of Mitochondria-Lipid Droplets Membrane Contact and Regulates Myocardial Lipid Metabolism

Lang Hu, Daishi Tang, Bingchao Qi, Dong Guo, Ying Wang, Jing Geng, Xiaoliang Zhang, Liqiang Song, Pan Chang, Wensheng Chen, Feng Fu and Yan Li**

Supplementary Materials

Detailed Methods

Conditional cardiac-specific Mfn2 Knockout Mice

All animal experimental procedures were approved by the Airforce Medical University Animal Use and Care Committee. The floxed-Mfn2 ($Mfn2^{fl/fl}$) mice were developed by Shanghai Biomodel Organism Science & Technology Development Lab (Shanghai, China). Mice expressing the cardiac α-myosin heavy chain (αMyHC)-Cre recombinase were purchased from Jackson Laboratory (Bar Harbor, ME, USA). The cardiac-specific male Mfn2 knockout ($Mfn2^{fl/fl}$Cre, $Mfn2^{CKO}$) mice were generated by crossing $Mfn2^{fl/fl}$ with (αMyHC)-MerCreMer Cre mice. Littermate male $Mfn2^{fl/fl}$ mice were considered as control mice. To induce Cre expression, mice were treated with tamoxifen (Sigma) by intraperitoneal injection once a day for 4 to 6 days at a dosage of 20 mg/kg per day from 4-weeks of age. Then tamoxifen-induced $Mfn2^{CKO}$ and $Mfn2^{fl/fl}$ mice were fed a libitum high-fat dite (HFD, 60 kcal% fat, Research Diets, D12492) and control diet (CD, 10 kcal% fat, Research Diets, D12450J) for 5 weeks. All mice (male) were C57BL/6J background and housed in temperature-controlled cages with a 12-hour light-dark cycle and given free access to water and food.

Construction design and generation of conditional cardiac-specific Mfn2 transgenic mice

Conditional cardiac-specific Mfn2 transgenic mice were generated with an LSL cassette under the control of a proximal CAG promoter followed by Mfn2 cDNA. Briefly, CAG-LSL-Mfn2-IRES-tdTomato-WPRE-polyA expression cassette were inserted at the site of Rosa26 gene through homologous recombination. Activation of (αMyHC)-MerCreMer Cre recombinase enable deletion of the floxed STOP cassette, thereby allowing conditional expression of Mfn2 in cardiomyocytes. Genotype were identified by PCR of tail DNA according to standard procedures. Resultant Tg (CAG-LSL-Mfn2-IRES-tdTomato-WPRE-polyA) $Cre^{+/-}$ transgenic mice ($Mfn2^{TG}$) were used for the experiments. Littermates without Cre-induced recombination were used as a control (NTG). Tamoxifen was intraperitoneally injected at 4-weeks of age to induce

Cre activity. Overexpression of Mfn2 in hearts was confirmed by RT-PCR and western-blotting. $Mfn2^{TG}$ and NTG mice were fed ad libitum HFD (60 kcal% fat, Research Diets D12492) and control diet (CD, 10 kcal% fat, Research Diets D12450J) for 10 weeks.

Chemicals and antibodies

Palmitate (#P0500), Nicotinamide (#47865-U), MG-132 (#SML-1135) and trichostatin A (#T1952) were purchased from Sigma-Aldrich (MO, USA). Bodipy 493/503, Bodipy 558/568 red C12, mito-tracker red, mito-tracker green were obtained from Thermo fisher (USA).

The primary antibodies are as follows: Mfn2 (#ab25236 and #ab56889, Abcam), Hsc70 (#ab51052, Abcam), IgG (#RIGG-69, Abcam), acetylated-lysine (#9441, Cell signaling technology), ubiquitin (#3936, Cell signaling technology), flag (#ab205606, Abcam), β-actin (#66009-1-Ig, Proteintech).

Secondary antibodies were as follows: Cy3–conjugated Affinipure Goat Anti-Rabbit IgG (#SA00009-2, Proteintech), Cy3–conjugated Affinipure Goat Anti-Mouse IgG (#SA00009-1, Proteintech), Fluorescein (FITC)–conjugated Affinipure Goat Anti-Rabbit IgG (#SA00003-2, Proteintech), Fluorescein (FITC)–conjugated Affinipure Goat Anti-Mouse IgG (#SA00003-1, Proteintech).

Echocardiography

Echocardiography was performed with a VEVO 3100 echocardiography system (VisualSonics Inc., Toronto, Canada) as previously described[1]. Generally, mice were anaesthetized with 2.5% isoflurane and anesthesia was maintained with 2% isoflurane. In order to rule out the influence of heart rate variation on cardiac function, all mice subjected to echocardiography are anesthetized suitably to maintain a heart rate of 400-450 times per minute. A real time ECG monitoring equipment were used to detect mice heart rate while performing echocardiography.

M-mode echocardiography was obtained to record the left ventricular systolic and diastolic motion profile. Doppler echocardiography was obtained to determine diastolic trans-mitral blood flow velocities for peak early (E) and late (A) fillings. All

the echocardiographic images were analyzed using Vevo 3100 software as previously described[1].

Biochemical and Anthropometric analysis in mice

Body weight and heart weight were measured in all mice. Low density lipoprotein (LDL)-cholesterol, high-density lipoprotein (HDL)-cholesterol and triglycerides were detected by clinical laboratory of Tangdu Hospital using a Chemray800 automatic biochemical analyzer (Rayto, Shenzhen, China) with standard protocol. Blood glucose level were measured with a sip-in sampling glucose meter (Glucometer 580, Yuwell, China).

Tissue harvesting

Mice were euthanized by CO_2. After chest cavity was opened, the heart was cut out and perfused with PBS. For molecular analysis, the organ was placed immediately in ice cold PBS to remove blood. Then the organ was conserved in snap-frozen in liquid nitrogen. For histological analysis, tissue was fixed in 4% paraformaldehyde solution. For transmission electron microscopy, heart samples from the left ventricular free wall were isolated and fixed with 2.5% glutaraldehyde in a 0.1 M phosphate buffer.

Transmission electron microscopy (TEM)

TEM analyze was performed as previously described[2]. All images were collected by a by a technician blinded to the treatment with one transmission electron microscope (JEM-1230, JEOL Ltd., Tokyo, Japan) at 300 kV. TEM images were analyzed by a technician blinded to the treatment using Image J software. The morphology of mitochondria and lipid droplet were analyzed as previously described[3]. The number of LDs combined with LDs diameter were used for the analysis of intramyocardial LDs accumulation. Percent of LDs contact with mitochondria and mean LDs-mitochondria contact length were calculated to reflect mitochondria-LDs contact as previously described[3]. For calculation of percentage of LDs with direct contact to mitochondria, 6 visual field was randomly selected and the LDs number was recorded. LDs which had direct contact with mitochondria refers to that LDs and mitochondria closely contacted with each other with no visible gaps were present. In contrast,

segregated LDs with no visible connection to the mitochondria was excluded. Then, the percentage of LDs with direct contact to mitochondria was calculated. All calculation and measurements were taken independently by one experienced technician who was blinded to the experimental design.

Lipidomics

For lipidomics, heart sample were collected and conserved in liquid nitrogen until lipid extraction. Lipidomics experiments were performed as described previously[4, 5]. samples were thawed on ice, and metabolites were extracted from 20 µL of each sample using 120 µL of precooled 50% methanol buffer. Then the mixture of metabolites was vortexed for 1 min and incubated for 10 min at room temperature, and stored at -20°C overnight. The mixture was centrifugated at 4,000 g for 20 min, subsequently the supernatant was transferred to 96-well plates. The samples were stored at -80°C prior to the LC-MS analysis using a TripleTOF 5600 Plus high-resolution tandem mass spectrometer (SCIEX, Warrington, UK) with both positive and negative ion modes. The acquired LC-MS data pretreatment was performed using XCMS software. The open access databases, KEGG and HMDB, were used to annotate the metabolites by matching the exact molecular mass data to those from the database within a threshold of 10 ppm.

Down-regulation and up-regulation of target genes

Empty adenoviral vectors (Ad-EV), adeno-associated virus 9 expressing Hsc70-specific small hairpin RNA (AAV-9 Hsc70-shRNA), recombinant adenoviral vectors expressing Mfn2 (Ad-Mfn2), Mfn2-specific small hairpin RNA (Ad-Mfn2 shRNA), Mfn1 (Ad-Mfn1), Opa1(Ad-Opa1) or Hsc70-specific small hairpin RNA (Ad-Hsc70 shRNA) were conducted by Hanbio Biotechnology Ltd (Shanghai, China). For adenovirus transfection, cardiomyocytes were incubated in Opti-MEM (Invitrogen) containing adenovirus (MOI: 50/1) for 6-8 h.

For construction of adenovirus encoding mitochondrial-targeted red fluorescent protein (peptides), the mitochondrial targeting elements of cox8 (mitochondrial targeting sequence), a mitochondrial outer membrane protein, was fused with Cy3-

labeled full-length or fragments of Mfn2 protein.

Intra-myocardial injection of adeno-associated virus (AAV)

For intramyocardial injection of AAV, mice were anaesthetized using 2.5% isoflurane, intubated and maintained under anesthesia with 2% isoflurane during the surgical procedure. After the hearts were exposed, AAV was injected (using a 50 μl needle, Hamilton, 705RN, USA) into the left ventricle free wall (10 μl at each of four sites).

Western blotting and quantitative real-time (RT)-PCR

Human atrial tissue, mice heart tissue and primary cardiomyocytes were analyzed by western-blotting as previously described[6]. Primary antibodies against the following proteins were used: β-actin (Proteintech, #20536-1-AP), Mfn2 (Abcam, #ab56889), Hsc70 (Abcam, #ab51052).

Total RNA was extracted with RNAisoPlus (Takara, Japan), cDNA was synthesized with a PrimeScript™ RT Reagent Kit with gDNA Eraser (Takara), and quantitative RT-PCR was performed with SYBR® Premix Ex Taq™ II (Takara) following the manufactures' protocols. Primers used in this study are all listed in Table S1.

Histological Analysis

Oil Red O staining were performed following standard procedures as previously described[7]. All steps were performed on the machine with Ventana solutions.

Primary culture of neonatal rat cardiomyocytes

Primary cardiomyocytes were prepared from neonatal rat hearts as previously described[1]. Thereafter, primary cardiomyocytes were subjected to control medium (Con) or control medium containing palmitate (Pal)[8].

Immunofluorescence microscopy

Cells grown on confocal dishes (NEST Biotechnology) were fixed with 4% paraformaldehyde for 20 min, permeabilized with 0.01% Triton X-100 in PBS, and blocked with 1% BSA in PBS. Cells were stained overnight with primary anti-Mfn2 (1:150, Abcam, #ab56889) and anti-Hsc70 (1:100, Abcam, #ab51052). Subsequently, samples were incubated with Alexa Fluor 555 or Alexa Fluor 488 or Delight 405 secondary antibodies respectively. A confocal laser-scanning microscope (Nikon A1

plus Confocal Microscope, Nikon, Japan) was used to capture immunofluorescence images.

Fluorescent imaging of fatty acids (FAs), LDs and mitochondria

For fatty acid pulse-chase, cells were incubated with culture medium containing 1uM Bodipy 558/568 C12 (Red C12, Life Technology, USA) for 16 hours. Cells were then washed 3 times with HBSS buffer before confocal imaging. Mitochondria were labeled by 100nM Mitotracker-red or Mitotracker-green (Life Technology, USA). To view LDs, Bodipy 493/503 (Life Technology) was added to cells at 200 ng/ml immediately before confocal detection. All fluorescent images were acquired with a confocal laser-scanning microscope (Nikon A1 plus Confocal Microscope, Nikon, Japan). For FAs-LDs or mitochondria overlap coefficient analysis, ImagJ Colocalization Analysis software (https://imagej.net/imaging/colocalization-analysis) was used to calculate the overlap coefficient. For tethering between LDs and mitochondria analysis, the percentage of mitochondrial signal connected to LDs signal versus total LD signal was calculated.

FAs transfer pulse-chase assay

Neonatal cardiomyocytes were incubated with control medium containing 300μM palmitate and 1μm Bodipy 558/568 red C12 for overnight. Palmitate were employed to induce LDs biogenesis while red C12 were used to label fatty acids. Cells were then washed three times with PBS and chased every 5 minutes for 30 minutes in HBSS.

LD isolation

LDs were isolated from mice heart according to the methods reported by Zhang *et al*[9, 10]. Specially formulated buffer A (25 mM tricine pH 7.6, 250 mM sucrose) and buffer B (20 mM HEPES, pH 7.4, 100 mM KCl and 2 mM MgC$_{l2}$) were prepared before isolation. Mice heart sample were transferred to a tube containing Buffer A with 0.2mM PMSF. Then the sample was homogenized with a p polytron homogenizer for 30s with a SpeedMill Plus homogenizer (Analytik Jena) for 25 times. The homogenates were centrifuged at 3000g for 30 min to remove the debris. Supernatant

with 3mL buffer B was loaded into an UltraClear centrifuge tube (Beckman Coulter). The tube was centrifuged at 288 000 g for 54 min at 4°C. Then, a light-yellow band containing pure LDs (See supplementary figures) on the top of the tube was collected.

Western blotting

Heart tissue of mouse and human myocardial specimens were lysed for immunoblotting (150 mmol/L sodium chloride, 50 mmol/L Tris, 1 mmol/L sodium fluoride, 1 mmol/L DTT, 1 mmol/L EDTA, 10 μg/μL leupeptin, 10 μg/μL aprotinin, 0.1 mmol/L sodium vanadate, 1 mmol/L PMSF, and 0.5% NP-40). Equal amounts (20 μg) of samples were subjected to SDS-PAGE gel for electrophoresis followed by semidry transfer onto Immobilon-P filter papers (Millipore, Billerica, USA). The membranes were blocked with 5% dry milk or 5% BSA in PBS-Tween buffer (0.1% Tween 20; pH 7.5) for 60 minutes and incubated with primary antibodies. Anti-rabbit and anti-mouse secondary antibodies were obtained from Proteintech (China). The immunoreactive bands were detected by digital chemiluminescence system (Bio-rad, Chemi-Doc XRS+). Related signals were quantified using the Imagelab software.

Co-immunoprecipitation

Co-immunoprecipitation (Co-IP) was performed using a Pierce Classic Magnetic IP/Co-IP Kit (Thermo Fisher Scientific, USA) according to the manufacturer's protocol as previously described[6]. Mice heart lysates were incubated with Mfn2 antibody (Abcam, #ab205236), Flag antibody (#ab205606, Abcam) or Hsc70 antibody ((#ab51052, Abcam) for immunoprecipitation. Western-blot analysis were further used to detect the efficiency of immunoprecipitation. For IP experiments, left ventricular tissues was collected for analysis. IP experiment were performed in triplicate and repeated independently three times. Proteins were immunoblotted with Mfn2 antibody (Abcam, #ab205236), Hsc70 antibody (#ab51052, Abcam), igG antibody (#RIGG-69, Abcam), GRP78 antibody (Abcam, #ab21685), and flag antibody (#ab205606, Abcam) respectively.

LC-MS/MS analysis

Liquid chromatography tandem mass spectrometry (LC-MS/MS) analysis was

performed using a Q Exactive HF-X mass spectrometer (Thermo Fisher Scientific) coupled with EASY-nLC 1200 UHPLC system (Thermo Fisher Scientific) at Novogene Genetics (Beijing, China) as previously described[6]. The 40 most abundant precursor ions from full MS scan were selected for analysis at a resolution of 15,000 (at 200 m/z). Raw files of mass spectrometry test were identified by MaxQuant (www.maxquant.org). Spectra data were searched refer to a UniProt mouse database (www.uniprot.org).

Proximity ligation assay (*in situ* PLA)

Proximity ligation assay was carried out following the instructions of Duolink In Situ Red Starter Kit Mouse/Rabbit (Sigma-Aldrich, DUO92101). Briefly, cells were fixed with 4% paraformaldehyde for 10 minutes at room temperature and then permeabilized for 5 minutes with 0.5% Triton X-100. The following steps were performed according to instructions of Duolink® Proximity Ligation Assay Kit. Cells were incubated with primary antibodies (Mfn2, 1:1000, Abcam; Hsc70, 1:1000, Abcam) at toom temperature for 1 hour. Images were captured with a Nikon A1R Scanning Laser Microscope.

Plasmid's construction and transfection.

Full length Mfn2 and specific-sequence Mfn2 were cloned to a Flag- tagged destination vectors for different detections. Point mutation for Mfn2 was conducted by site-directed mutagenesis. Plasmid transfection in 293T cell line were conducted with Lipofectamine 3000 Transfection Reagent (Invitrogen) according to the manufacturer's instructions.

Human iPSC-derived cardiomyocytes

Human iPSC-derived cardiomyocytes (hiPSC-CMs) were provided by Cellapy Biotechnology Co., Ltd (Cellapy: CardioEasy®CA2001106, Beijing, China). HiPSC-CMs were incubated in maintaining medium (Cellapy: CardioEasy®CA 2015002, Beijing, China) at 37 ℃, 5% CO_2, with culture medium refreshed every 2 days.

Study population

Obese patients (BMI≧30) and non-obese age-matched controls were continuously

recruited at the Department of Cardiology, Tangdu hospital and Department of Cardiac Surgery, Xi’an GaoXin Hospital, Xi’an, China. Patients with any of the following conditions were excluded: I) Severe cardiomyopathy (i.e. clinical signs of heart failure, LVEF<50%, left atrial dilatation (>40 mm), systolic pulmonary artery pressure >40 mm Hg, or N-terminal pro-B-type natriuretic peptide level > 300 pg /ml); II) Atrial fibrillation/ atrial flutter or history of atrial fibrillation.

Clinical and echocardiographic data were recorded at the time of admission. Right atrial tissue was collected during right atrium cannulation from 3 obese patients and 3 non-obese patients who undergoing coronary artery bypass grafting (CABG). Samples were immediately prepared for molecular analysis. The study protocol was approved by Local Ethics Committee (IEC of Institution for National Drug Clinical Trails, Tangdu Hospital, Fourth Military Medical University, No.202103-12). All participants were fully aware of the investigational nature of the study and provided written informed consent for their participation.

Supplementary reference

1. Hu L, Ding M, Tang D, Gao E, Li C, Wang K, Qi B, Qiu J, Zhao H, Chang P, Fu F, Li Y. Targeting mitochondrial dynamics by regulating Mfn2 for therapeutic intervention in diabetic cardiomyopathy. Theranostics 2019;**9**(13):3687-3706.
2. Ding M, Hu L, Yang H, Gao C, Zeng K, Yu M, Feng J, Qiu J, Liu C, Fu F, Li Y. Reduction of SIRT1 blunts the protective effects of ischemic post-conditioning in diabetic mice by impairing the Akt signaling pathway. Biochim Biophys Acta Mol Basis Dis 2019;**1865**(6):1677-1689.
3. Boutant M, Kulkarni SS, Joffraud M, Ratajczak J, Valera-Alberni M, Combe R, Zorzano A, Cantó C. Mfn2 is critical for brown adipose tissue thermogenic function. Embo j 2017;**36**(11):1543-1558.
4. Nguyen TB, Louie SM, Daniele JR, Tran Q, Dillin A, Zoncu R, Nomura DK, Olzmann JA. DGAT1-Dependent Lipid Droplet Biogenesis Protects Mitochondrial Function during Starvation-Induced Autophagy. Dev Cell 2017;**42**(1):9-21.e5.

5. Benjamin DI, Li DS, Lowe W, Heuer T, Kemble G, Nomura DK. Diacylglycerol Metabolism and Signaling Is a Driving Force Underlying FASN Inhibitor Sensitivity in Cancer Cells. ACS Chem Biol 2015;**10**(7):1616-23.
6. Qi B, He L, Zhao Y, Zhang L, He Y, Li J, Li C, Zhang B, Huang Q, Xing J, Li F, Li Y, Ji L. Akap1 deficiency exacerbates diabetic cardiomyopathy in mice by NDUFS1-mediated mitochondrial dysfunction and apoptosis. Diabetologia 2020;**63**(5):1072-1087.
7. Zhao H, Zhang F, Sun D, Wang X, Zhang X, Zhang J, Yan F, Huang C, Xie H, Lin C, Liu Y, Fan M, Yan W, Chen Y, Lian K, Li Y, Zhang L, Wang S, Tao L. Branched-Chain Amino Acids Exacerbate Obesity-Related Hepatic Glucose and Lipid Metabolic Disorders via Attenuating Akt2 Signaling. Diabetes 2020;**69**(6):1164-1177.
8. Tsushima K, Bugger H, Wende AR, Soto J, Jenson GA, Tor AR, McGlauflin R, Kenny HC, Zhang Y, Souvenir R, Hu XX, Sloan CL, Pereira RO, Lira VA, Spitzer KW, Sharp TL, Shoghi KI, Sparagna GC, Rog-Zielinska EA, Kohl P, Khalimonchuk O, Schaffer JE, Abel ED. Mitochondrial Reactive Oxygen Species in Lipotoxic Hearts Induce Post-Translational Modifications of AKAP121, DRP1, and OPA1 That Promote Mitochondrial Fission. Circ Res 2018;**122**(1):58-73.
9. Zhang H, Wang Y, Li J, Yu J, Pu J, Li L, Zhang H, Zhang S, Peng G, Yang F, Liu P. Proteome of skeletal muscle lipid droplet reveals association with mitochondria and apolipoprotein a-I. J Proteome Res 2011;**10**(10):4757-68.
10. Hernández-Albaladejo I, Ruíz-Palacios M, Gázquez A, Blanco JE, Parrilla JJ, Larqué E. A method for lipid droplet isolation from human placenta for further analyses in clinical trials. Acta Obstet Gynecol Scand 2014;**93**(11):1198-202.

Supplementary Tables

Table S1 Primers used for real time PCR.

**Primer**	**Sequence**
**β-actin Forward**	5’-GTC CCT CAC CCT CCC AAA AG-3’

**β-actin Reverse**	5’-GCT GCC TCA ACA CCT CAA CCC-3’
**mfn2 Forward**	5’-CTT GAA GAC ACC CAC AGG AAC A-3’
**mfn2 Reverse**	5’-GGC CAG CAC TTC GCT GAT AC-3’
**ATGL Forward**	5’-CGC CTT GCT GAG AAT CAC CAT-3’
**ATGL Reverse**	5’-AGT GAG TGG CTG GTG AAA GGT-3’
**CPT1b Forward**	5’-TAG GAC AGG CAG AAA ATT G-3’
**CPT1b Reverse**	5’-CAG TAG GAG CCG ATT CAA AA-3’
**PPARα Forward**	5’-AGG AAG CCG TTC TGT GAC AT-3’
**PPARα Reverse**	5’-TTG AAG GAG CTT TGG GAA GA-3’
**CPT2 Forward**	5’-AGC CAG TTC AGG AAG ACA GA-3’
**CPT2 Reverse**	5’-GAC AGA GTC TCG AGC AGT TA-3’
**CD36 Forward**	5’-TGT GTT TGG AGG CAT TCT CA-3’
**CD36 Reverse**	5’-TGG GTT TTG CAC ATC AAA GA-3’

Table S2 Mass spectrum analysis of Mfn2-interacting proteins.

Table S3 Mass spectrum analysis of LD proteins.

**Protein name**	**Score ranking**
**Hspd1**	1
**Plin5**	2
**GRP78**	3
**Ldha**	4
**Pdia6**	5

**Protein name**	**Score ranking**
[illegible]	[illegible]
**Dlat**	2
[illegible]	[illegible]
**Nexn**	4
[illegible]	[illegible]
**Pdhx**	6
**Hsc70**	**7**
**Eef2**	8
**GRP78**	**9**
**60S ribosomal protein L4**	10
**DEAD-box helicase 3, X-linked**	11
**Dihydrolipoyl dehydrogenase**	12
**Caldesmon**	13
**Annexin A6**	14
**HSP90**	15

**Rpsa**	6
**Hsc70**	7
**Catalase**	8
**Tpp2**	9
**Uba1**	10
**Vinculin**	11
**Cpt-1b**	12
**RTN4**	13
**Plin2**	14
**Rab7**	15

Table S4

Demographics, laboratory parameters, medications, and echocardiographic data in obese and non-obese patients.

	Non-obese (n=16)	Obese (n=13)	P value
**Demographics**			
**Age (years)**	59.7±5.6	56.6±11.0	0.34
**Gender (F:M)**	12: 4	10: 3	0.90
**BMI (kg/m^2)**	23.1±2.1	32.9±3.17	<0.01*
**Systolic blood pressure (mmHg)**	127.7±14.9	139.3±27.9	0.16
**Diastolic blood pressure (mmHg)**	78.3±10.1	86.5±11.7	0.06
**Laboratory values**			
**FPG (mmol/L)**	5.34±1.08	5.66±0.75	0.37
**Triglycerides (mmol/L)**	1.55±0.57	2.34±0.41	<0.01*
**HDL-C (mmol/L)**	0.94±0.16	0.88±0.21	0.35
**LDL-C (mmol/L)**	2.05±0.83	1.97±0.62	0.76
**Total cholesterol (mmol/L)**	3.69±1.12	4.54±0.81	<0.01*

**Medications**			
**Statins (%)**	12.5	84.6	<0.01*
**ACE-i/ARBs (%)**	31.2	46.2	0.41
**Beta-blockers (%)**	12.5	15.4	0.76
**Diuretics (%)**	6.3	15.4	0.42
**Cardiac Function**			
**LVEDD**	50.1±8.3	53.4±3.7	0.20
**LVESD**	33.7±9.8	37.2±4.5	0.26
**EF (%)**	64.8±5.1	58.1±6.6	<0.01*
**FS (%)**	35.6±3.6	30.1±4.2	<0.01*
**E/A**	1.1±0.4	0.8±0.2	0.03*
**SV (ml)**	70.4±13.0	78.3±10.4	0.09
**LVPW**	9.6±1.1	10.5±1.1	0.06
**IVS**	9.9±1.1	11.4±1.3	0.03*
**LAD**	37.3±5.4	41.7±5.5	0.04*

Values are expressed as Mean± SEM. P values refer to Student t and Chi^2 tests. BMI, body mass index; FPG, fasting plasma glucose; HDL-C, high density lipoprotein cholesterol; LDL-C, low density lipoprotein cholesterol; ACE-i, angiotensin converting enzyme inhibitors, ARBs, angiotensin receptor blockers. LVEDD, left ventricular end-diastolic diameter; LVESD, left ventricular end-systolic diameter; EF, ejection fraction; FS, fractional shortening; E/A, ratio of mitral peak velocity of early filling (E) to mitral peak velocity of late filling (A); SV, stroke volume; LVPW, left ventricular posterior wall; IVS, inter ventricular septum; LAD, left atrial diameter.

Supplementary Figures

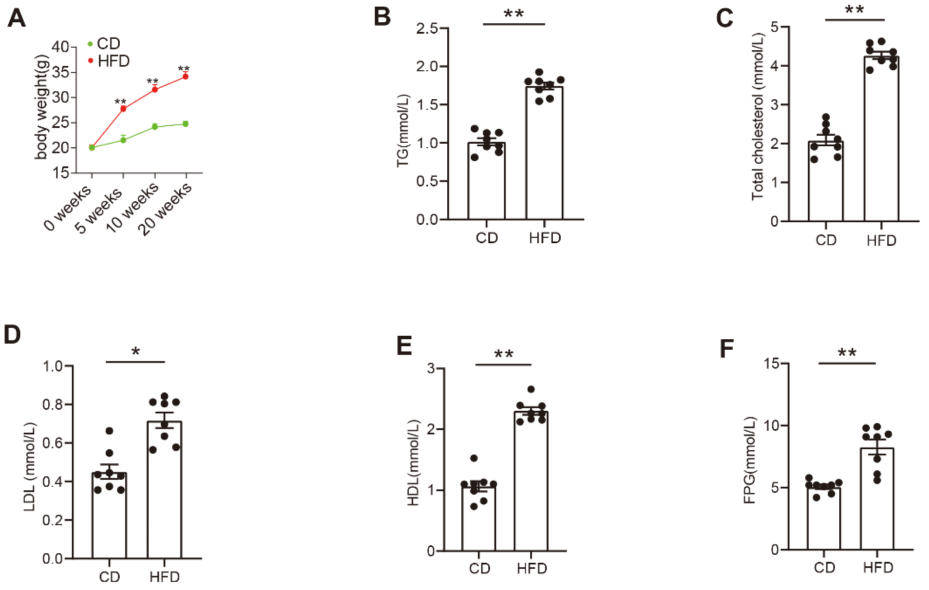

Figure S1 Serum lipid level and fasting plasma glucose level of CD- and HFD-fed mice. A, Body weight of CD- and HFD-fed mice at different time points. n=8 mice per group. **B,** Fast plasma glucose level. **C,** Plasma triglycerides level. **D,** Plasma total cholesterol level. **E,** High-density lipoprotein level. **F,** Low-density lipoprotein level. Mean±SEM, n=8 mice per group, **P* < 0.05, ***P* < 0.01.

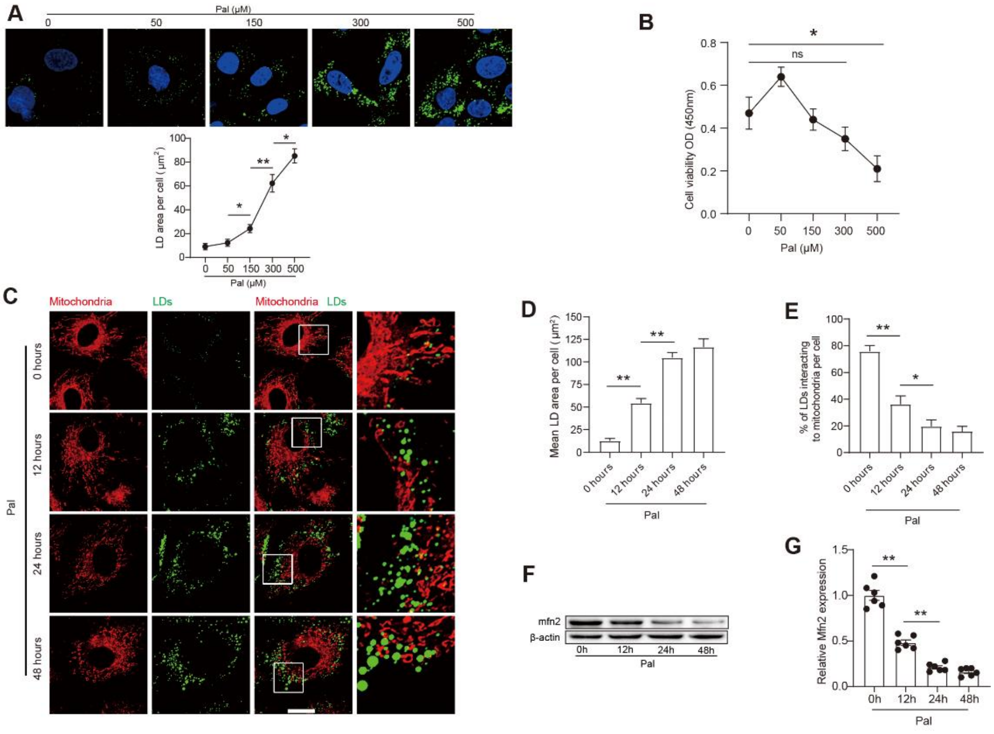

Figure S2 Mfn2 protein level were determined and MLC were detected by labeling mitochondria (red) and LDs (green) respectively in palmitate-treated cardiomyocytes. A&B, Cardiomyocytes were treated with palmitate at different concentrations, then LDs were labeled with Bodipy 493/503 (green) and cell viability was examined by CCK8 assay. **C**, Cardiomyocytes were treated with palmitate, then LDs were labeled with Bodipy 493/503 (green) and mitochondria was labeled with Mito-tracker Red (red) at time points indicated. **D,** LDs area per cell (Mean± SEM, n=5 independent experiments, 30 cells were quantified per group, ***P*<0.01). **E,** Percentage of LDs with direct contact to mitochondria was measured (Mean± SEM, n=5 independent experiments, 30 cells were quantified per group, ***P*<0.01). **F&G**, Mfn2 protein level were determined by Western-bolt at different time point of palmitate treating.

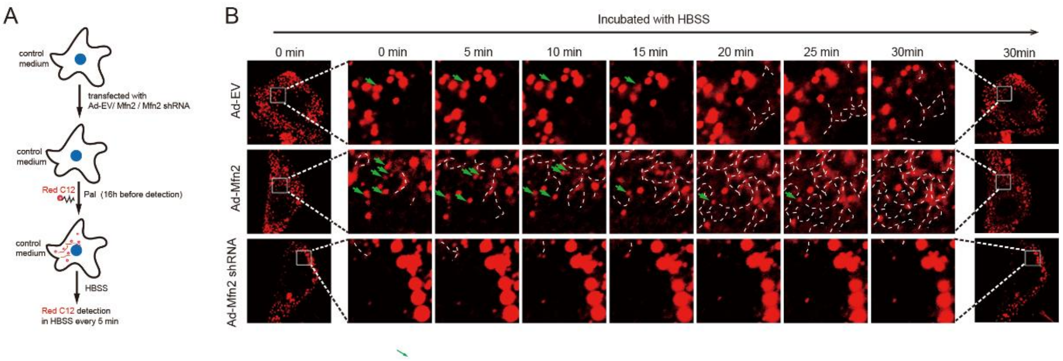

Figure S3, A Red C12 pulse-chase assay were employed to further determine the traffic of FAs from LDs to mitochondria. A, Schematic representation of the red C12 pulse-chase assay to visualize FAs transport from LDs to mitochondria. B, Red C12 Pulse-chase assay showing the intracellular transport of FAs. Degraded LDs during detection are visible as bright spots (green arrow) and mitochondria are visible as faint lines (white dashed lines).

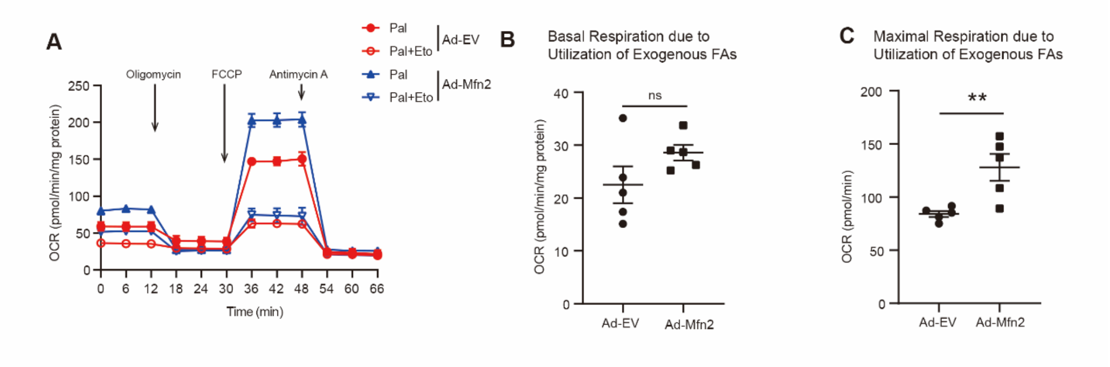

Figure S4 Mfn2 overexpression promoted lipid oxidation in mitochondria. **A,** Oxygen consumption rate (OCR) in the presence or absence of etomoxir. **B,** Basal respiration due to utilization of exogenous FAs. **C,** Maximal respiration due to utilization of exogenous FAs. Data presented as mean ± SEM, n=4 independent experiments. Differences are significant for **P<0.01.

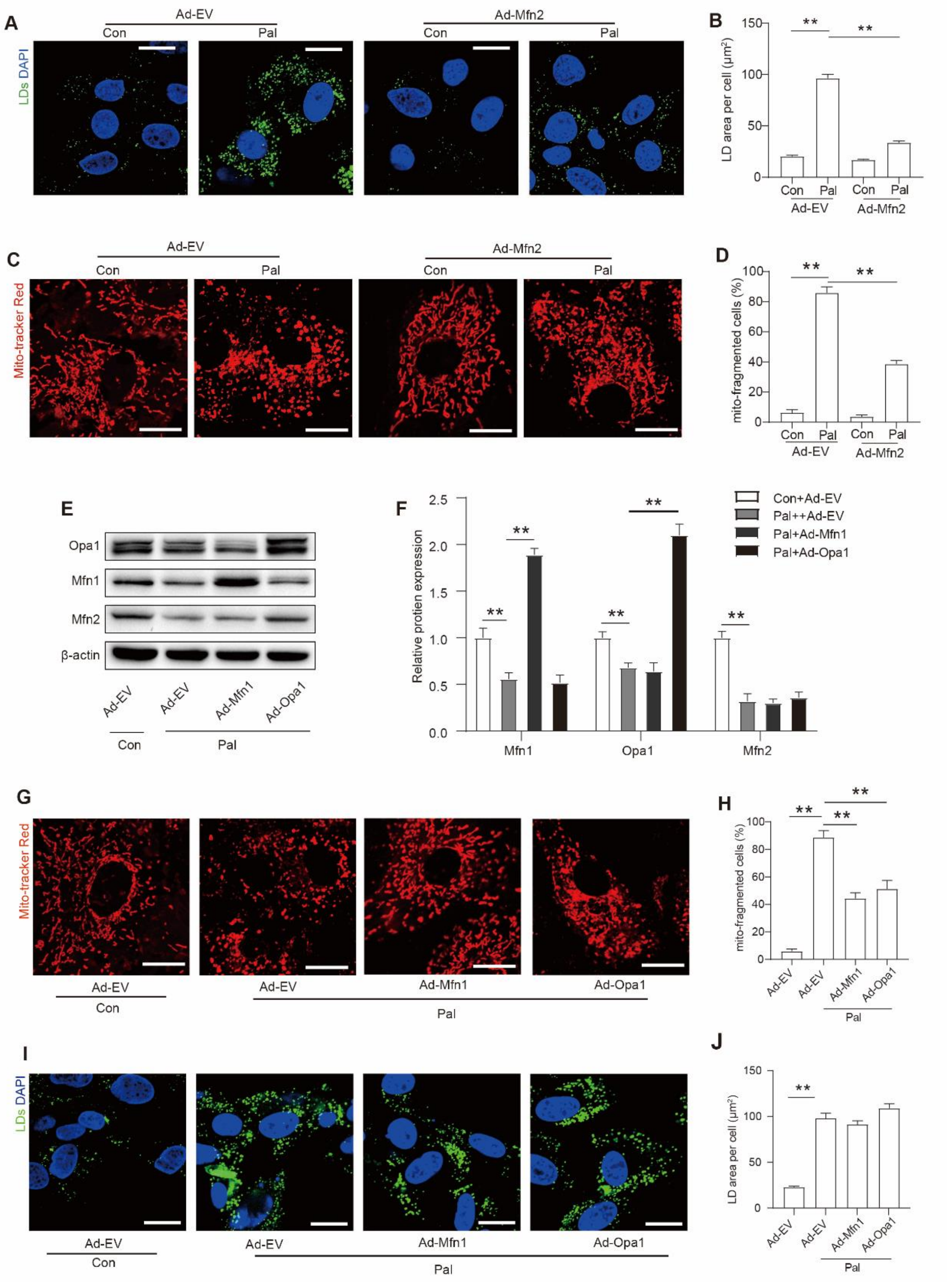

Figure S5 Mfn2 prevents LDs accumulation in palmitate-treated primary cardiomyocytes. A, Control- or palmitate- treated primary cardiomyocytes were transfected with adenovirus expressing Mfn2 (Ad-Mfn2) or control adenovirus (Ad-EV), then LDs were labeled with Bodipy 493/503 (green dots). Original

magnification ×600. **B,** Average LD numbers per cell (Mean± SEM, n=5 independent experiments, 30 cells were quantified per group, **P<0.01). **C&D,** Mitochondrial morphology were determined using Mito-tracker Red and mito-fragmented cells percentage were measured in each group (Mean± SEM, n=5 independent experiments, 30 cells were quantified per group, **P<0.01). **E&F,** Representative blot images and quantitative analysis of Mfn1, Mfn2 and Opa1 (Mean± SEM, n=4 independent experiments, **P<0.01). **G&H,** Adenovirus expressing Mfn1 or Opa1 were used to promote mitochondrial fusion in primary cardiomyocytes in presence of palmitate. LDs were then labeled and counted (Mean± SEM, n=5 independent experiments, 30 cells were quantified per group, **P<0.01). **I&J,** Mitochondrial morphology were determined using Mito-tracker Red and mito-fragmented cells percentage were measured in each group. (Mean± SEM, n=5 independent experiments, 30 cells were quantified per group, **P<0.01).

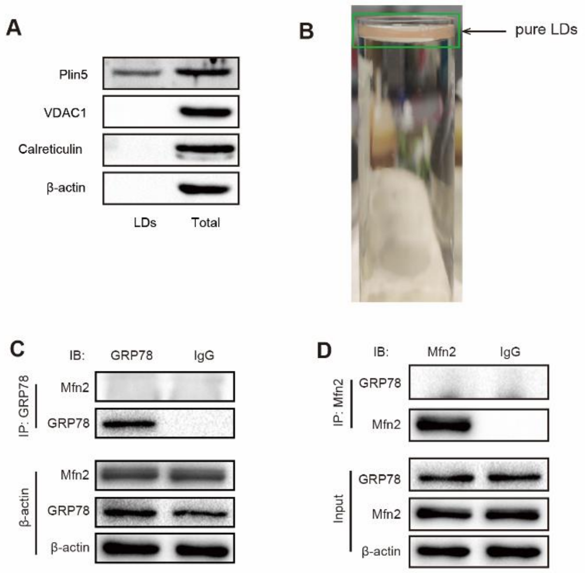

Figure S6 Lipid droplet were perfectly isolated and interaction of GRP78 and Mfn2 were detected. A, Lipid droplets were identified by Western-blot analysis. VDAC1, marker of mitochondria; Calreticulin, marker of ER; β-actin, marker of

cytosolic protein; Plin5, marker of LDs. **B,** Photo of LDs isolated from mice hearts. **C&D,** GRP78 and Mfn2 failed to pull-down each other in the IP experiments. n=4 independent experiments.

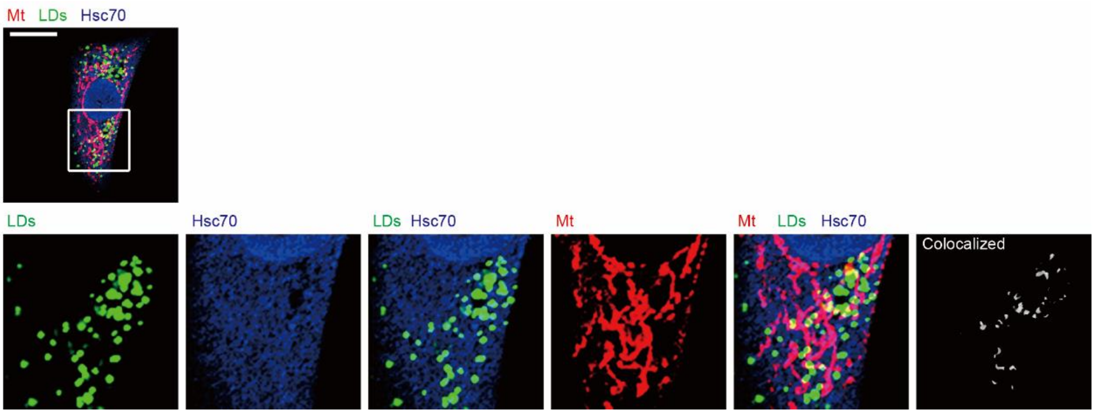

Figure S7 Hsc70, LDs and mitochondria co-localized in primary cardiomyocytes. Original magnification ×600.

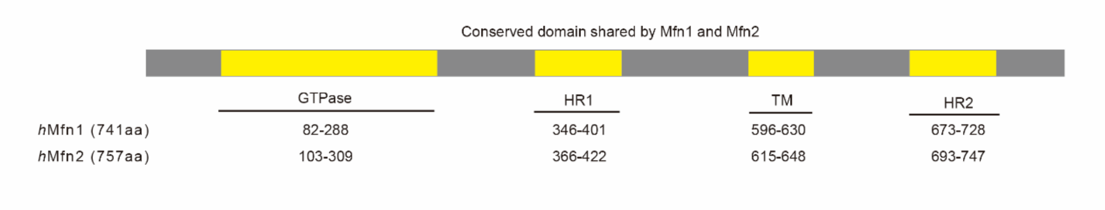

Figure S8, Schematic diagrams of conserved domains shared by Mfn1 and Mfn2.

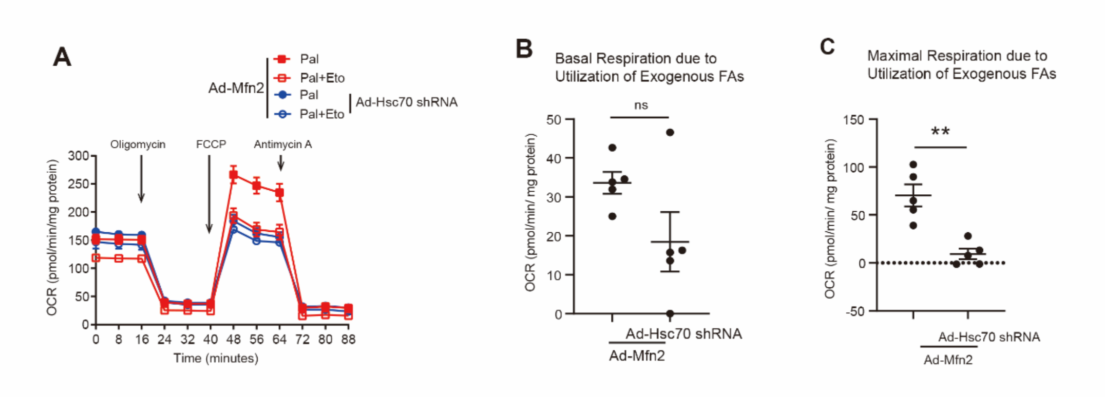

Figure S9 Mfn2-driven mitochondrial lipid oxidation were blunted by Hsc70 knockdown. **A,** Oxygen consumption rate (OCR) in the presence or absence of etomoxir. **B,** Basal respiration due to utilization of exogenous FAs. **C,** Maximal respiration due to utilization of exogenous FAs. Data presented as mean ± SEM, n=4

independent experiments. Differences are significant for **P<0.01.

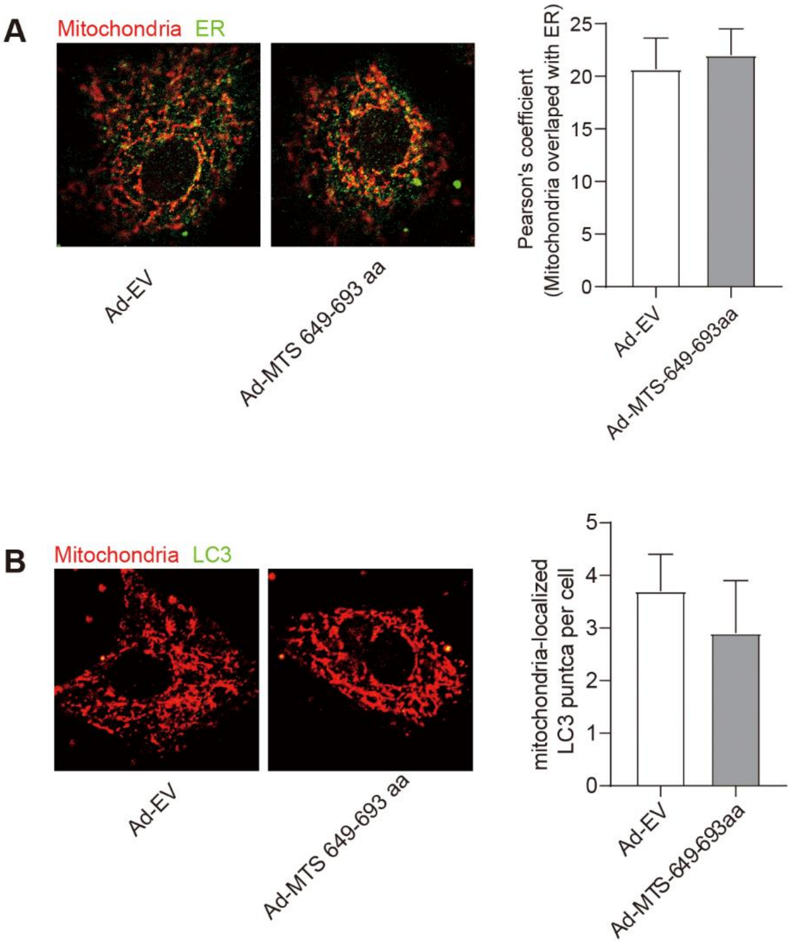

Figure S10 Ad-MTS-649-693aa failed in inducing mitophagy and mitochondria-SR tethering in the cardiomyocytes. A, Mitochondria and SR were labeled with red and green (Calreticulin) fluorescence respectively, and the relative co-efficient was analyzed. B, Mitophagy was determined by LC3 puncta (green) colocalized with mitochondria (red).

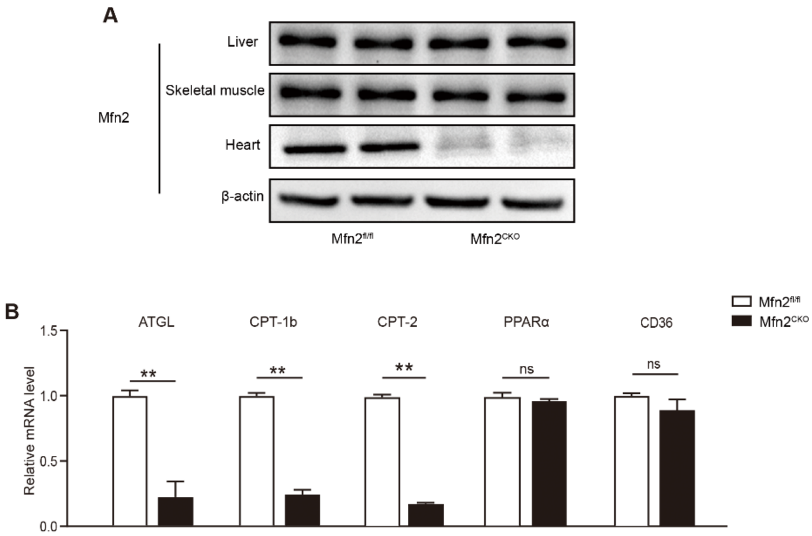

Figure S11 Cardiac-specific knockout of Mfn2 in Mfn2^{CKO} mice was confirmed by western-bolt and RT-PCR analysis. A, Representative blot images of Mfn2 protein level in different tissue from Mfn2$^{fl/fl}$ and Mfn2^{CKO} mice (n= 4 mice per group). **B,** mRNA level of gene involved in lipolysis (PPARα, ATGL, CPT-1b, CPT-2) and lipid uptake (CD36) were determined by RT-PCR (n= 8 mice per group). Data presented as Mean ± SEM. Differences are significant for **P<0.01. ns, no significance.

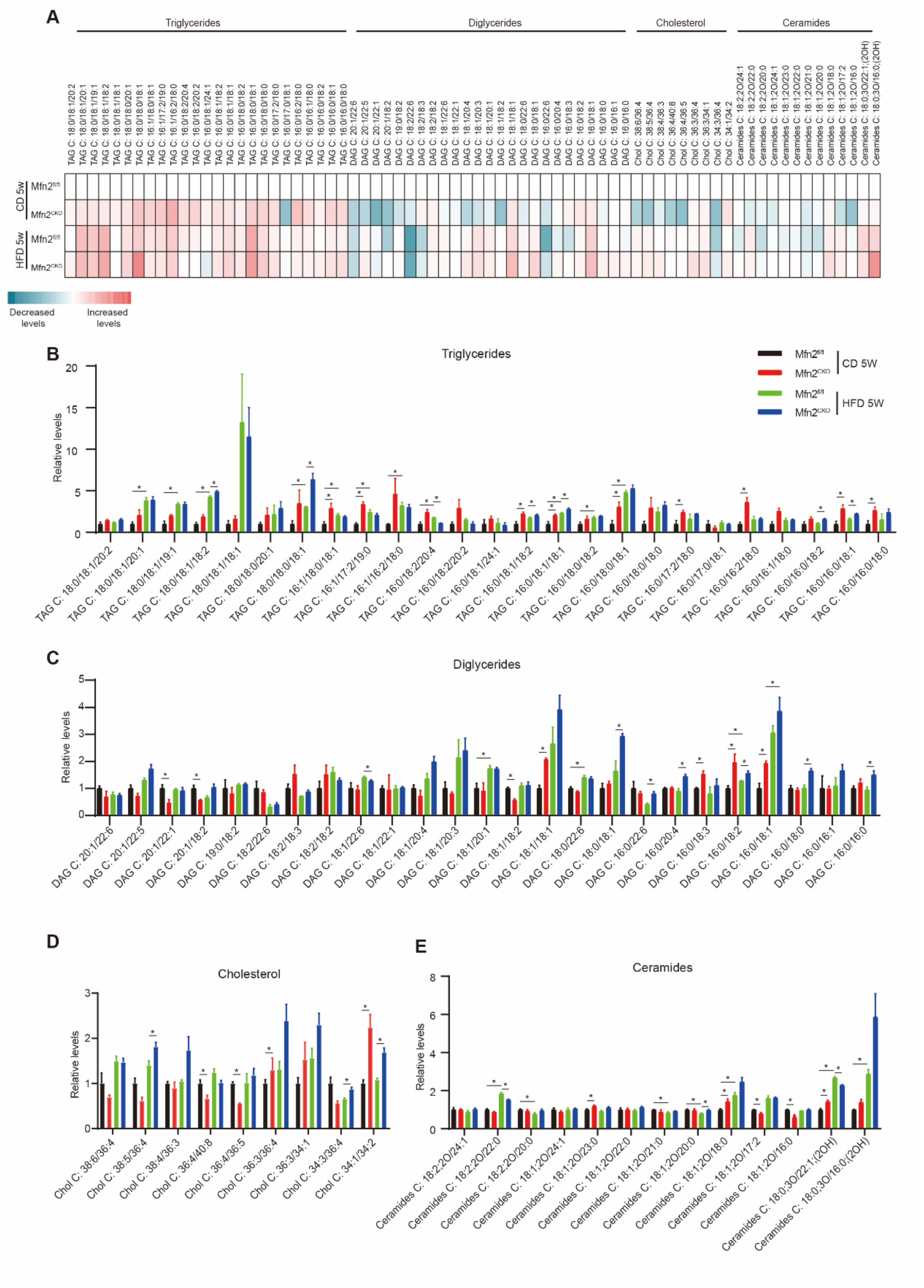

Figure S12 Mass spectrometry (MS)-based quantification of cardiac lipid species across the different experimental groups. A, Heatmap of metabolic alterations organized by lipid class. **B-E,** Quantification showing the relative levels of lipids in 4 four groups indicated. Data presented as Mean ± SEM, n=3 mice per group.

Differences are significant for *P<0.05.

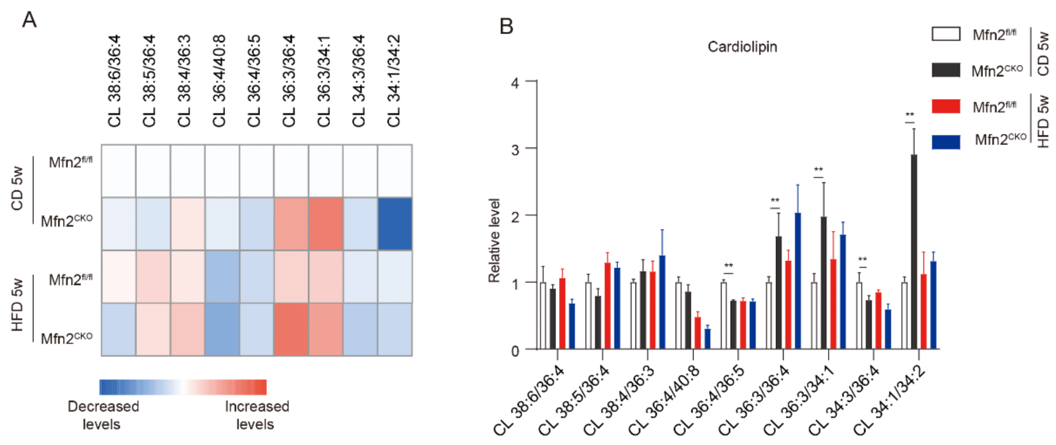

Figure S13. Cardiac cardiolipin level was determined in heart of Mfn2CKO mice. A-B, Heatmap of cardiolipin and quantification showing the relative levels of cardiolipin in 4 four groups indicated.

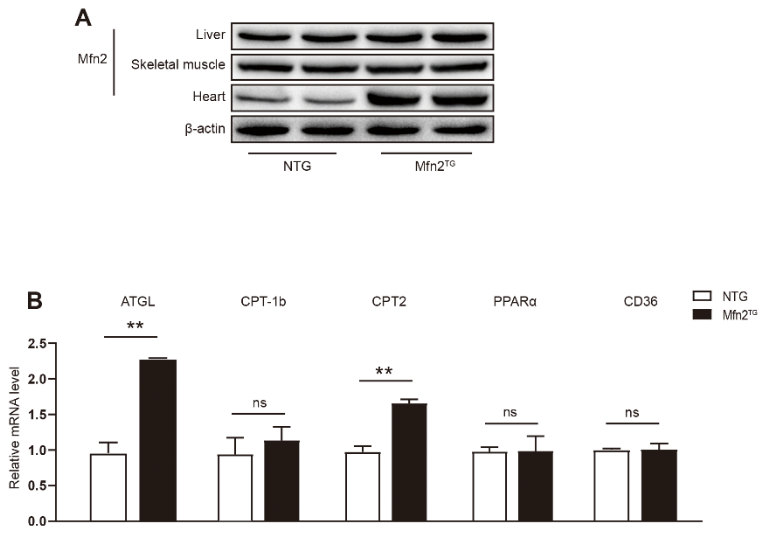

Figure S14 Cardiac-specific transgene of Mfn2 was confirmed by western-bolt and RT-PCR analysis. **A,** Representative blot images of Mfn2 protein level in different tissue from NTG and Mfn2^{TG} mice (n=4 mice per group). **B,** mRNA level of gene involved in lipolysis (PPARα, ATGL, CPT-1b, CPT-2) and lipid uptake (CD36) were determined by RT-PCR (n=8 mice per group). Data presented as Mean ± SEM.

Differences are significant for **P<0.01. ns, no significance.

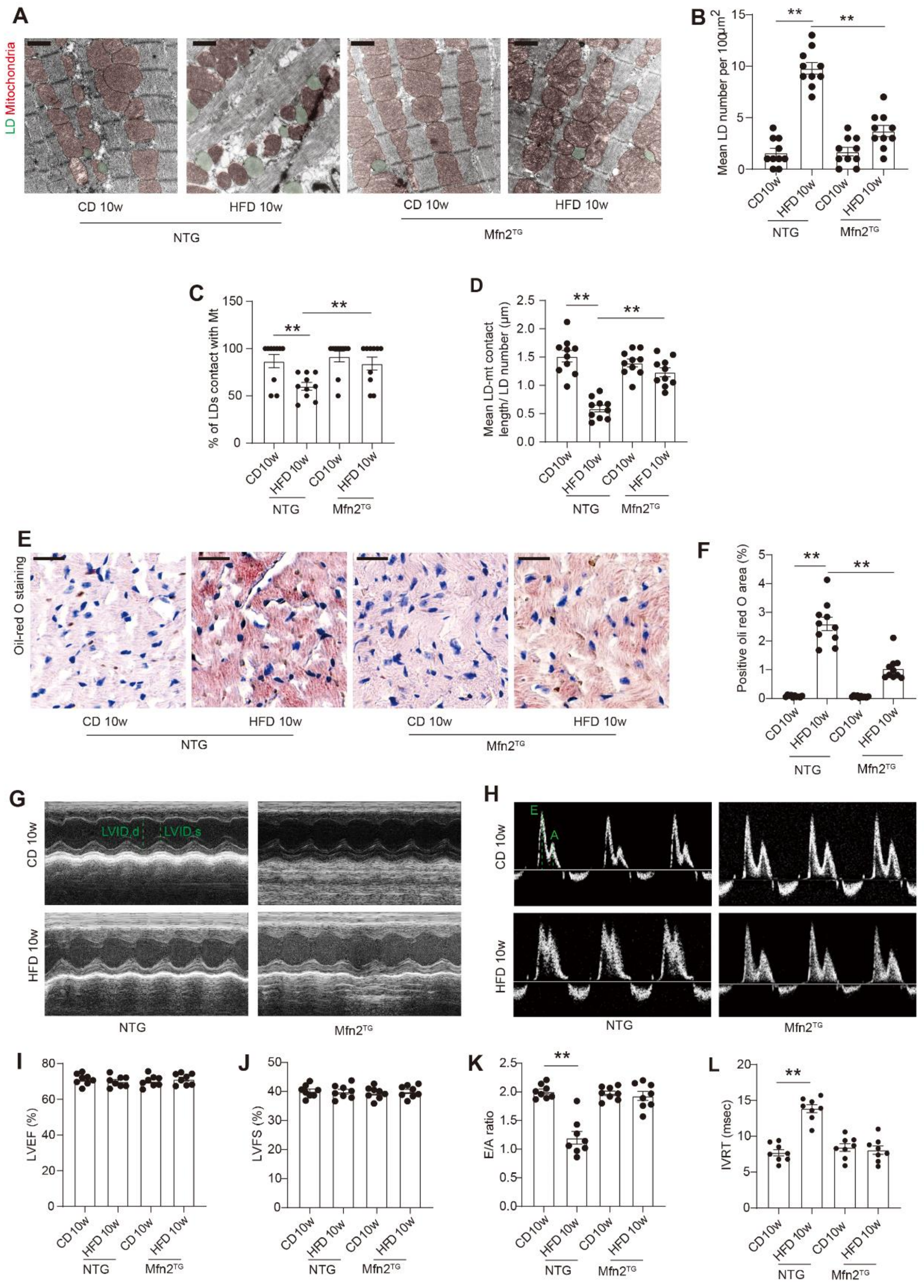

Figure S15 In-vivo Mfn2 overexpression prevents HFD-induced mitochondria-LDs disassociation and reduced intra-myocardial lipid accumulation. A,

Representative TEM images of mitochondria and LDs from hearts of NTG and Mfn2^{TG} mice after 10 weeks' CD or HFD feeding. LDs are labeled as green while mitochondria are labeled as red. Scale bars, 1μm. **B,** Intra-myocardial LD number were calculated to assess lipid accumulation in myocardium (Mean± SEM, n=10 images from 8 mice each group, **P<0.01). **C,** Percentage of LDs contact with mitochondria (Mean± SEM, n=10 images from 8 mice each group, **P<0.01). **D,** Mean LD-mitochondria contact length were measured to reflect LD-mitochondria tethering in myocardium. (Mean± SEM, n=10 images from 8 mice each group, **P<0.01). **E**, Oil-red O staining showing intra-myocardial lipid content, oil-red O positive area are shown as red, scale bar=25μm. **F,** Quantitative analysis of positive Oil-red O area in each group (Mean± SEM, n=6 mice each group, **P<0.01). **G-L,** Echocardiographic assessment were performed on NTG and Mfn2^{TG} mice after 10 weeks' CD or HFD feeding. n=8 mice per group. **G,** Representative images of M-mode echocardiography. **H,** Representative Doppler flow measurement of mitral inflow. **I-L,** left ventricle ejection fraction (LVEF), left ventricle fractional shortening (LVFS), E/A ratio and isovolumic relaxation time (IVRT). (Mean± SEM, **P<0.01). CD, chow diet; HFD, high-fat diet; LD, lipid droplet; Mt, mitochondria.

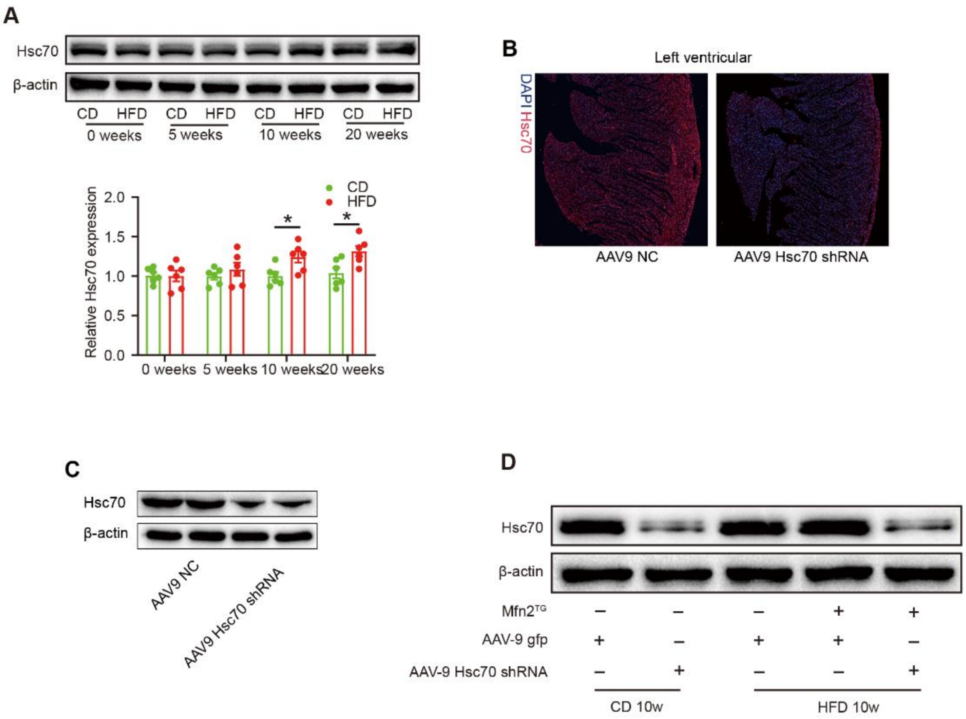

Figure S16 Hsc70 were knocked-down by AAV-9 injection in mice heart. A, Representative blot images and quantitative analysis of Hsc70 protein level in mice heart as HFD-fed prolonged. **B&C,** Hsc70 in the hearts of WT mice subjected to AAV9 Hsc70 shRNA injection were determined by immunofluorescence (IF) staining and Western-blot analysis. **D,** Hsc70 knockdown induced by AAV-9 Hsc70 shRNA intramyocardial injection in mice hearts were determined by Western-blot analysis. Data presented as Mean ± SEM. n=8 mice per group. Differences are significant for *P<0.05.

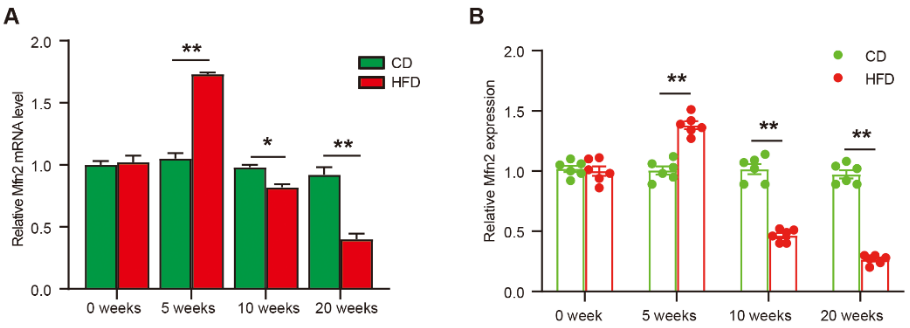

Figure S17 Mfn2 protein level and mRNA level were quantified in hearts of HFD-fed mice at different time points. Data presented as mean ± SEM, n=6 mice per group. Differences are significant for **P<0.01, *P<0.05.

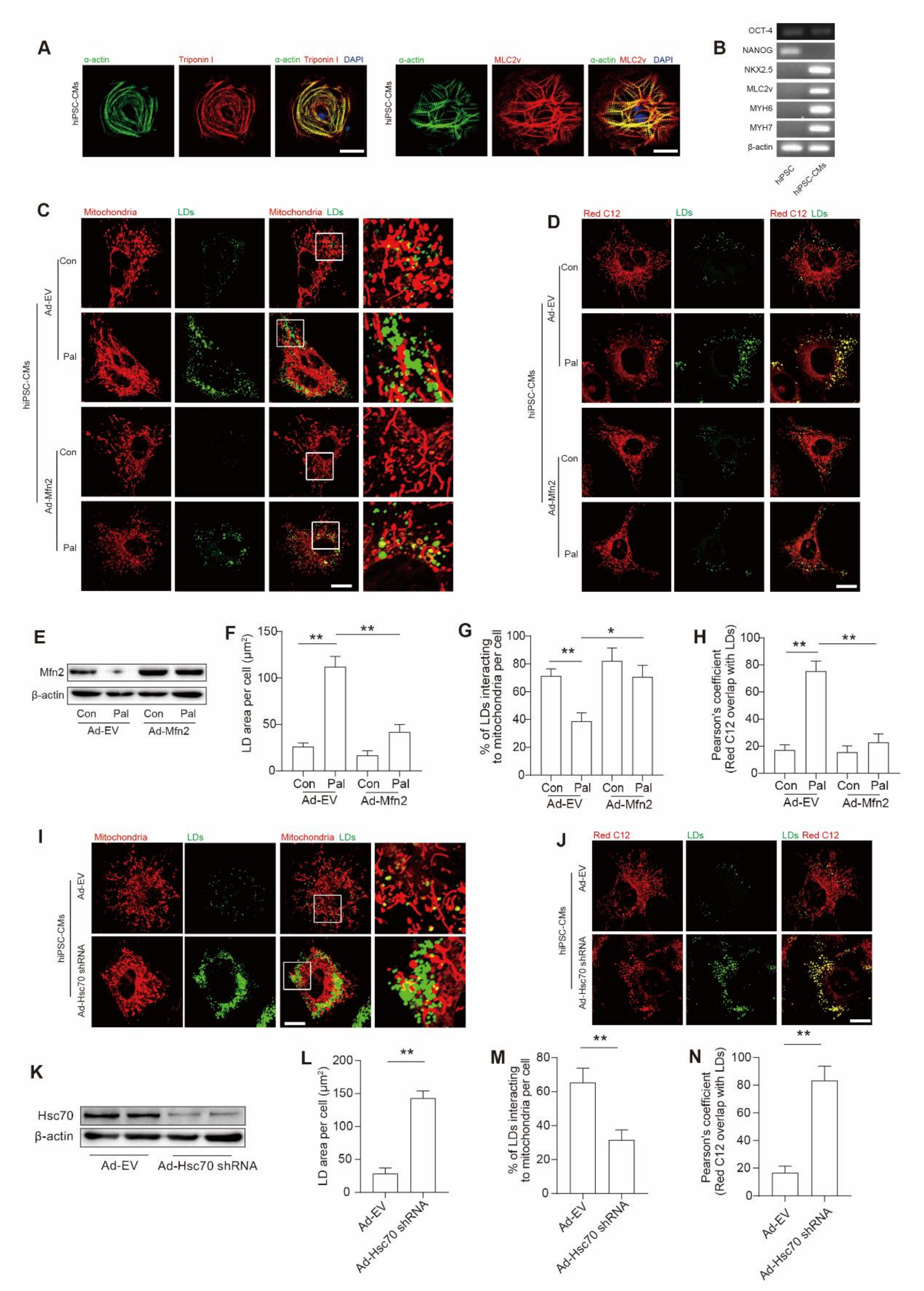

Figure S18 Mfn2-Hsc70 mediated MLC and lipid accumulation in palmitate-treated hiPSC-CMs. A-B, Cardiac ontogeny of hiPSC-CMs was confirmed by analyses of cardiac cell-specific transcriptional, structural, and functional markers. **A,** Immunostaining for sarcomeric α-actinin, Troponin I and MLC2v in hiPSC-CMs. **B,** RT-PCR analysis of the cardiac-specific transcription factors (NKX2.5) and structural genes (MLC2v, MYH6, and MYH7) in hiPSC-CMs. **C,** Control- or Pal treated hiPSC-MCs were transfected with adenovirus expressing Mfn2 (Ad-Mfn2), then LDs were labeled with Bodipy 493/503 (green) and mitochondria was labeled with Mito-tracker Red (red). Original magnification ×600. **D,** hiPSC-MCs were treated as in **C**, then LDs (green) and FAs (Red C12, red) were labeled respectively. **E,** Mfn2 protein level were determined by Western-blot in palmitate-treated hiPSC-CMs after Ad-Mfn2 or Ad-EV transfection. **F**, LDs area per cell (Mean± SEM, n=5 independent experiments, 30 cells were quantified per group, $**P<0.01$). **G,** Percentage of LDs with direct contact to mitochondria was measured (Mean± SEM, n=5 independent experiments, 30 cells were quantified per group, $**P<0.01$). **H,** Relative localization of Red C12 with LDs was measured in cells treated as in E (Mean± SEM, n=5 independent experiments, 30 cells were quantified per group, $**P<0.01$). **I,** hiPSC-MCs were transfected with adenovirus expressing Hsc70 shRNA (Ad-Hsc70 shRNA), then LDs were labeled with Bodipy 493/503 (green) and mitochondria was labeled with Mito-tracker Red (red). Original magnification ×600. **J,** Hsc70 protein level were determined by Western-blot in palmitate-treated hiPSC-CMs after Ad-Hsc70 shRNA transfection. **K,** hiPSC-MCs were treated as in I, then LDs (green) and FAs (Red C12, red) were labeled respectively. **L,** LDs area per cell (Mean± SEM, n=5 independent experiments, 30 cells were quantified per group, $**P<0.01$). **M,** Percentage of LDs with direct contact to mitochondria was measured (Mean± SEM, n=5 independent experiments, 30 cells were quantified per group, $**P<0.01$). **N,** Relative localization of Red C12 with LDs was measured in cells (Mean± SEM, n=5 independent experiments, 30 cells were quantified per group, $**P<0.01$).
